# Mechanical Regulation of Mitochondrial Dynamics and Function in a 3D-Engineered Liver Tumor Microenvironment

**DOI:** 10.1021/acsbiomaterials.2c01518

**Published:** 2023-03-31

**Authors:** Adam Frtús, Barbora Smolková, Mariia Uzhytchak, Mariia Lunova, Milan Jirsa, Yuriy Petrenko, Alexandr Dejneka, Oleg Lunov

**Affiliations:** † Department of Optical and Biophysical Systems, Institute of Physics of the Czech Academy of Sciences, Prague 18221, Czech Republic; ‡ Institute for Clinical & Experimental Medicine (IKEM), Prague 14021, Czech Republic; § Department of Neuroregeneration, Institute of Experimental Medicine of the Czech Academy of Sciences, Prague 14220, Czech Republic

**Keywords:** engineered cell microenvironments, cancer, mitochondria, mechanical forces, extracellular matrix, cytoskeleton, cell plasticity

## Abstract

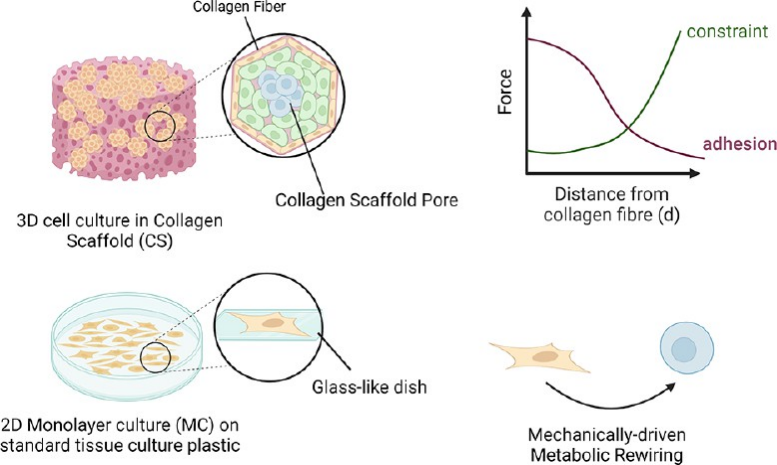

It has become evident that physical stimuli of the cellular microenvironment transmit mechanical cues regulating key cellular functions, such as proliferation, migration, and malignant transformation. Accumulating evidence suggests that tumor cells face variable mechanical stimuli that may induce metabolic rewiring of tumor cells. However, the knowledge of how tumor cells adapt metabolism to external mechanical cues is still limited. We therefore designed soft 3D collagen scaffolds mimicking a pathological mechanical environment to decipher how liver tumor cells would adapt their metabolic activity to physical stimuli of the cellular microenvironment. Here, we report that the soft 3D microenvironment upregulates the glycolysis of HepG2 and Alexander cells. Both cell lines adapt their mitochondrial activity and function under growth in the soft 3D microenvironment. Cells grown in the soft 3D microenvironment exhibit marked mitochondrial depolarization, downregulation of mitochondrially encoded cytochrome *c* oxidase I, and slow proliferation rate in comparison with stiff monolayer cultures. Our data reveal the coupling of liver tumor glycolysis to mechanical cues. It is proposed here that soft 3D collagen scaffolds can serve as a useful model for future studies of mechanically regulated cellular functions of various liver (potentially other tissues as well) tumor cells.

## Introduction

Various 3D cell culture platforms are becoming increasingly popular in studies aiming to better mimic *in vivo* physiological and pathophysiological conditions.^1−3^ Emerging evidence suggests that such platforms show improved capabilities of inducing *in-vivo*-like cell fates for distinct physiological and pathophysiological process research.^1−3^ Specifically, engineered 3D extracellular-matrix-based scaffolds hold great promises in producing relevant 3D mechanical environments to study the pathophysiological development of hepatocellular carcinoma (HCC) and providing more reliable *in vitro* models.^4−6^ In fact, the liver extracellular matrix (ECM) is a dynamic microenvironment that faces substation remodeling and changes in its stiffness upon injury and various pathological conditions.^7−9^ Furthermore, the stiffness of liver ECM modulates the development and progression of HCC, showing a stiffness value greater than that of a healthy liver.^10,11^ Values of liver stiffness around the range of 2.5–5.5 kPa are considered to be normal.^12,13^ On the contrary, both the benign and malignant liver tumors show higher than normal liver stiffness in the range of 10–75 kPa.^10,11,14,15^

Current research indicates that a stiffer ECM possesses strong modulatory effects on hepatocellular carcinoma cells, e.g., accelerates migration of cells,^16^ promotes proliferation and chemotherapeutic resistance,^5,6,17^ triggers epithelial–mesenchymal transition, and facilitates HCC metastasis.^18^ Currently, the majority of research that is focused on developing engineered mechanical environments to study HCC utilizes a stiff ECM with stiffness about 10–500 kPa.^6,18−20^ Little attention is given to study how substrates with stiffness lower than 1 kPa impact HCC cellular functions. However, it is known that HCC shows morphologic intratumoral heterogeneity.^21^ Moreover, developed and metastatic tumor tissues possess a high degree of mechanical heterogeneity bearing stiffer edges compared to the core of the tumor.^22,23^ Current research suggests that benign tumor tissues show uniform stiffness, whereas malignant tissues have stiffness heterogeneity with a prominent low-stiffness peak.^23^ Importantly, invasive cancer was shown to have 0.57 kPa stiffness.^23^ In fact, soft microenvironments of 0.1 kPa promote cancer chemoresistance.^24^ Furthermore, a soft HCC environment with 1 kPa stiffness triggers reversible cellular dormancy and stem cell characteristics in liver tumor cells.^17^ Indeed, tumor cell dormancy largely contributes to the maintenance of disseminated tumor cells in distant organs and delayed development of metastases.^25,26^ ECM remodeling was shown to be crucial in sustaining the dormancy of disseminated tumor cells.^27^ However, how ECM-derived mechanical cues regulate tumor cell metabolism and support dormancy of HCC cell is largely unknown. It is worth noting that apart from the stiffness of the cell microenvironment, current research focuses on the comparison of cellular responses in 2D and 3D cultures.^1,28−32^ Indeed, 3D culturing systems allow one to generate models for cellular culturing that possess physical cues lacking in 2D conditions.^1,28−32^ It becomes evident that 3D culturing results in the alteration of metabolic activity and signaling cascades in cancer cells.^1,28−32^ However, the knowledge on whether there are qualitatively and quantitatively different cellular responses in 2D vs 3D cultures remains rather fragmented.

**Figure 1:**
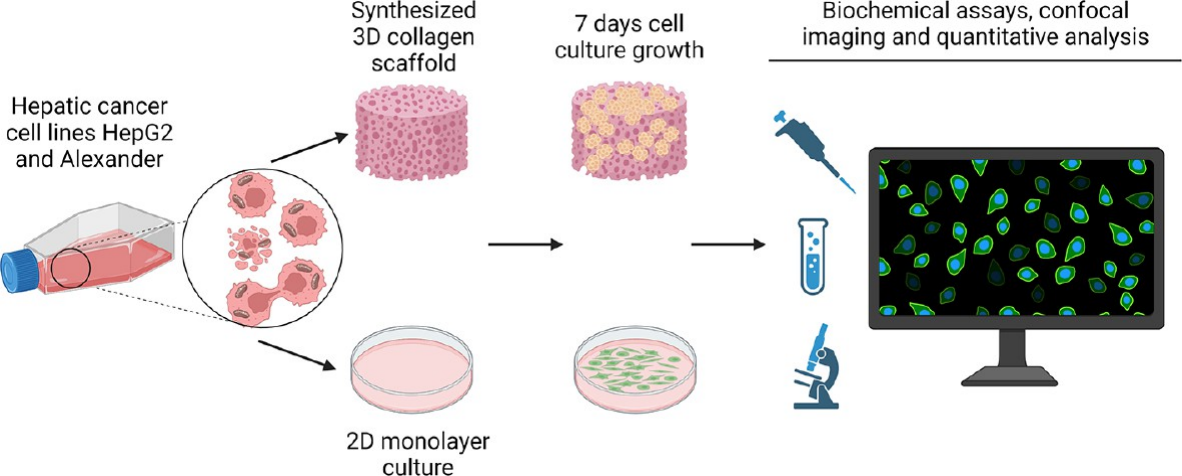
The schematic concept of the study. Alexander and HepG2 cells grown either in a standard monolayer culture (MC) or in collagen scaffolds (CSs). Cancer cell lines (HepG2 and Alexander) cultivated in the *in-house* produced CS biomaterial. CSs provided mechanical constraints for cellular proliferation: fibers and pores. Mechanical constraints arising from CSs causing mechanically driven metabolic rewiring. Created with BioRender.com.

We previously showed that culturing liver tumor cells in soft 3D substrates (stiffness ∼0.1 kPa) profoundly slows down cellular proliferation in a Yes-associated protein 1 (YAP)–mammalian target of Rapamycin (mTOR)-mediated manner.^33^ Further, a recent study indicated that the cellular microenvironment mechanically regulates glycolysis.^34^ Therefore, we hypothesized that mechanically modulated metabolic rewiring of liver tumor cells should be linked to the proliferation and dormant state of cell. We designed soft 3D collagen scaffolds to study the functional activity of liver tumor cells in a pathological mechanical environment (Figure 1). Here, we report that the soft 3D collagen scaffolds cause metabolic rewiring of liver tumor cells resulting in the upregulation of glycolysis. This mechanically modulated glycolysis upregulation is associated with mitochondrial depolarization, marked downregulation of mitochondrially encoded cytochrome *c* oxidase I, and slow proliferation of liver tumor cells.

## Materials and Methods

### Chemicals and Antibodies

We summarized all chemicals, fluorescent probes, antibodies, and TaqMan probes used in this study in Tables S1–S4 (Supporting Information), including dilutions, manufacturers’ names, and catalogue numbers.

### Synthesis and Characterization of Collagen Scaffolds

We synthesized porous collagen scaffolds (CSs) utilizing the cryostructuring technique described previously.^33^ Briefly, the lyophilized collagen (VUP Medical, Brno, Czech Republic) was dissolved in acetic acid at 4 °C. Afterward, the sample was centrifuged at 2000*g* for 30 min at 4 °C. The final collagen concentration was 8 mg/mL. A pasta-like sediment was poured into plastic cavities (3 mm diameter) and frozen at −20 °C for 24 h. Then, samples were immersed into precooled 96% ethanol with shaking for 48 h. Afterward, scaffolds were relocated into precooled 1% *N*-(3-dimethylaminopropyl)-*N*′-ethylcarbodiimide (EDC) hydrochloride (Sigma Aldrich, St. Louis, MO, USA) ethanol solution precooled to −20 °C and incubated for 24 h. Ultimately, after thawing, CSs were washed with ethanol and distilled water and then stored in 70% EtOH prior to cell seeding. To avoid a cytotoxic reaction, before cell seeding, CSs were extensively washed with phosphate-buffered saline (Thermo Fisher Scientific, Waltham, MA, USA).

We extensively characterized scaffolds in terms of viscoelastic properties, porosity, and diffusion previously.^33^ To thoroughly characterize the collagen scaffold microstructure in a liquid environment, we utilized the ColF fluorescent probe (Immuno Chemistry Technologies, Davis, CA, USA). ColF is a low-molecular-weight fluorescent probe that displays affinity for collagen.^33^ For deep morphological analysis of collagen fibers and pores that are created by collagen fiber organization, we utilized a high-resolution ultrafast confocal imaging system. Image analysis, quantification, and 3D reconstruction were done utilizing ImageJ (NIH, Bethesda, MD, USA) and the open-source software Icy (https://icy.bioimageanalysis.org).^35^

### Cell Lines and 3D Culturing

We used the human hepatoblastoma HepG2 cell line (American Type Culture Collection, ATCC, Manassas, VA, USA) and human hepatocellular carcinoma cell line Alexander (PLC/PRF/5, ATCC, Manassas, VA, USA) in this study. Cell cultures were cultivated in Minimum Essential Medium Eagle (BioConcept Ltd., Switzerland) supplemented with 10% fetal bovine serum (FBS, Thermo Fisher Scientific, US), 1% l-glutamine 100× (200 mM stock, Serana Europe GmbH, Germany), and 1% penicillin/streptomycin (Thermo Fisher Scientific, US). Cell cultures were kept in a humidified 5% CO_2_ atmosphere at 37 °C. The culture medium (EMEM) was changed once per week. Cells were regularly checked for common culture contamination such as mycoplasma using the MycoAlert Detection Assay (Lonza, Switzerland). All cell lines were authenticated by short tandem repeat (STR) DNA profiling (ATCC, Manassas, VA, USA).

Approximately 10^5^ HepG2 and Alexander cells in a volume of 30 μL were seeded into porous collagen scaffolds. CSs were placed in a 12-well plate. After 1 h, the fresh medium was added to cover the whole surface of CSs. For all utilized assays in the study, cells were cultivated in CSs for 7 days in a humidified 5% CO_2_ incubator at 37 °C, and the culture medium was changed every other day. Cells seeded on a standard glass-bottom dish (Cellvis, Mountain View, CA, USA) with a 35 mm diameter served as the monolayer culture (MC) model.

### Cell Proliferation Analysis

To determine cell proliferation dynamics, we stained the cells with the anti-Ki67 antibody and calculated the Ki-67 labeling index. Cells (Alexander and HepG2) were cultured in CS and MC models for 7 days. Afterward, cells were fixed with 4% paraformaldehyde in PBS (pH 7.4) at room temperature for 10 min. Samples were permeabilized using 0.5% Triton X-100 before staining. Immunofluorescence staining was performed on fixed cells using the anti-Ki67 antibody followed by the Alexa Fluor 568-conjugated secondary antibody (Thermo Fisher Scientific, Waltham, MA, USA). Dilutions and catalogue numbers of the primary antibodies used are given in Table S3. Hoechst 33342 dye was used to counterstain nuclei to assess the total cell number. Stained cells were imaged using the spinning disk confocal microscope IXplore SpinSR (Olympus, Tokyo, Japan). The ImageJ software (NIH, Bethesda, MD, USA) was used for image processing and quantification. The Ki-67 labeling index (%) was calculated as follows: Ki-67 positive nucleus/total cell number × 100. Any definite Ki67 nuclear staining was considered positive. Ki67 positivity was classified as positive or negative based on pixel color and intensity in accordance with ref (36). In total, we assessed *n* = 20–39 randomly selected fields per condition out of four independent experiments to achieve reliable statistical sampling. In the case of 3D CSs, we acquired confocal 3D optical sectioning for each randomly selected field and performed averaged cell counting in five optical sections per filed.

### Cell Extracts and Immunoblot Analysis

Analysis of protein expression in samples was done using the semiquantitative technique of immunoblot analysis. We utilized a radioimmunoprecipitation (RIPA) buffer (Millipore, Burlington, VT, USA) for rapid and efficient cell lysis and solubilization of proteins following manufacturer instructions and our verified protocol.^37−39^ To ensure the equal loading of proteins, we determined the total protein concentration in cell lysates using the Micro BCA Protein assay Kit (Thermo Fisher Scientific, Waltham, MA, USA) according to the manufacturer instructions. Subsequently, samples of whole-cell lysates containing an equal amount of protein (1 mg/mL) were prepared. Protein samples were separated using SDS-PAGE and then transferred to PVDF membranes. Blocking of membranes was achieved with 5% (w/v) nonfat dried milk or alternatively with 5% (w/v) BSA for 1 h. Then, PVDF membranes were incubated with various specific primary antibodies summarized in Table S3 at 4 °C overnight, washed in TBST, and incubated with the respective HRP-conjugated secondary antibody (Table S3) for 1 h. Finally, the chemiluminescence signal was revealed utilizing the imaging system G:BOX CHEMI XRQ (Syngene, Synoptics Group, Cambridge, UK).^37,38,40^ The acquisition software GeneTools (Syngene, UK) for chemiluminescence reading was used. Densitometric quantification of the bands’ intensity was done utilizing the GeneTools quantification software (Syngene, Synoptics Group, Cambridge, UK).

### Determination of Pyruvate Content

To monitor basic metabolic events, we utilized a Pyruvate Assay Kit (Sigma Aldrich, St. Louis, MO, USA) following the manufacturer’s guidelines. Briefly, we used cell extract samples obtained using the RIPA buffer. We determined the protein concentration using the Micro BCA Protein Assay Kit (Thermo Fisher Scientific, Waltham, MA, USA). To avoid any cross-reaction of pyruvate with lactate dehydrogenase (LDH) or other enzymes, all protein samples were deproteinized with a 10 kDa molecular weight cutoff (MWCO) spin filter (VIVASPIN; Sartorius, Goettingen, Germany). Processed samples and prepared standards were pipetted into 96-well plates (Eppendorf, Hamburg, Germany). Finally, the master reaction mix, consisting of the pyruvate assay buffer, pyruvate probe solution (5× diluted), and pyruvate enzyme mix, was added to samples. After 30 min of incubation at room temperature, fluorescence was captured. The intensity of fluorescence was measured using the Tecan microplate reader SpectraFluor Plus (Tecan, Männedorf, Switzerland). The pyruvate in the samples was calculated by referring to the calibration curve using standard pyruvate and expressed in nmol/mg of total protein content units.

### Determination of Lactate Content

Another metabolic marker that we examined was lactate utilizing the Lactate Assay Kit (Sigma Aldrich, St. Louis, MO, USA) in accordance with the manufacturer’s guidelines. Briefly, similarly to the pyruvate assay, we collected protein samples using the RIPA buffer, determined the concentration using the Micro BCA Protein Assay Kit, deproteinized samples with 10 kDa MWCO, and ran the fluorescence assay. The intensity of fluorescence was measured using the Tecan microplate reader SpectraFluor Plus (Tecan, Männedorf, Switzerland). The lactate in the samples was calculated by referring to the calibration curve using standard lactate and expressed in μmol/mg of total protein content units.

### Immunofluorescence Analysis

Cells (HepG2 and Alexander cell lines) were grown in either the monolayer culture (MC) or collagen scaffolds (CSs) under regular culturing conditions, i.e., humidified atmosphere with 5% CO_2_ at 37 °C. Afterward, cells were fixed using 4% paraformaldehyde solution in PBS (pH 7.4) at room temperature for 10 min. Permeabilization of cellular membranes was done using 0.5% Triton X-100 prior to the staining. Immunofluorescence staining was performed on fixed samples utilizing primary antibodies against specific proteins (Table S3) and the Alexa Fluor 568-conjugated secondary antibody (Thermo Fisher Scientific, Waltham, MA, USA). Stained cells were imaged using the spinning disk confocal microscope IXplore SpinSR (Olympus, Tokyo, Japan). Digital images were processed and quantified utilizing either the ImageJ software (NIH, Bethesda, MD, USA) or open-source software Icy (https://icy.bioimageanalysis.org).^35^

### RNA Isolation and Real-Time PCR

Total RNA from cell cultures grown in either the monolayer culture (MC) or collagen scaffolds (CSs) and human liver samples was isolated utilizing the RNeasy Mini Kit (Qiagen, Hilden, Germany) followed by DNA removal using the RNase-Free DNase Set (Qiagen, Hilden, Germany). The integrity and quantity of isolated RNA were checked by the Nanodrop One instrument (Thermo Fisher Scientific, Waltham, MA, USA). Subsequently, cDNA was generated using the Maxima H Minus First Strand cDNA Synthesis Kit (Thermo Fisher Scientific, USA). We utilized 2 μg of RNA to synthesize cDNA according to a previously published protocol.^41^

Further, we performed quantitative real-time PCR on an Applied Biosystems Viia7 Real Time PCR system (Applied Biosystems, Waltham, MA, USA) utilizing the Fast Advanced TaqMan Gene Expression Master Mix (Thermo Fisher Scientific, Waltham, MA, USA) and specific TaqMan gene expression assays (Table S4). Data were assessed using the MS Excel and MaxStat Pro 3.6 software (MaxStat, Cleverns, Germany). The expression of the target gene was normalized to *GAPDH* expression utilizing the 2^–ΔΔCT^ methodology previously published in ref (42).

### Laser System and High-Fluence, Low-Power (HFLP) Laser Treatment

To induce profound mitochondrial dysfunction, we irradiated cells with 649 nm wavelength HFLP utilizing a previously published laser system.^43,44^ We showed previously that continuous irradiation of cell with 649 nm HFLP with irradiance of <1 kW/cm^2^ leads to mitochondrial damages and subsequent cell death.^43,44^ The HFLP laser system characteristics and schematics of the experimental setup are shown on Figure S3. Briefly, 649 nm laser light from the system was delivered to the cell culture utilizing a fiber taper with a waist diameter of 15 ± 5 μm. The output laser power was kept at 63 ± 1 μW, which was measured by the optical power meter PM100D (Thorlabs, Newton, NJ, USA).^43,44^ Cells were seeded into collagen scaffolds (CSs), cultivated for 7 days, and labeled before laser irradiation. To detect the viability of cultured cells, we utilized a two-color fluorescence assay (LIVE/DEAD Viability/Cytotoxicity Kit; Thermo Fisher Scientific, Waltham, MA, USA). The optical taper was treated with 70% ethanol for disinfection before positioning to the cells. Establishing the position of the optical taper in close proximity to the cellular population was achieved using a Micromanipulator 5171 (Eppendorf, Wesseling-Berzdorf, Germany) that was connected to an Olympus IX83 microscope (Olympus, Tokyo, Japan).

### High-Resolution Spinning Disk Confocal Microscopy

For high-quality confocal images and 3D and 4D analysis, we used the brand-new high-resolution spinning disk confocal system IXplore SpinSR (Olympus, Tokyo, Japan).^45,46^ Cells (HepG2 and Alexander cell lines) were grown in either the monolayer culture (MC) or collagen scaffolds (CSs) under regular culturing conditions, i.e., humidified atmosphere with 5% CO_2_ at 37 °C. Afterward, cells were labeled with specific fluorescent probes; see details in Table S2. Alternatively, cells were fixed using 4% paraformaldehyde, permeabilized with 0.5% Triton X-100, and labeled with primary antibodies against specific proteins (Table S3) and the Alexa Fluor 568-conjugated secondary antibody (Thermo Fisher Scientific, Waltham, MA, USA). The confocal imaging system setup consists of the following functional units: an inverted microscope (IX83; Olympus, Tokyo, Japan) and a spinning disc confocal unit (CSUW1-T2S SD; Yokogawa, Tokyo, Japan). Fluorescence digital images were acquired via either a 20× objective LUCPLFLN20XPH NA 0.45 air lens (Olympus, Tokyo, Japan) or 100× silicone immersion objective UPLSAPO100XS NA 1.35 WD 0.2 silicone lens (Olympus, Tokyo, Japan). To excite fluorophores, the following lasers were utilized: 405 nm (50 mW), 488 nm (100 mW), and 561 nm (100 mW) laser diodes. Confocal digital images were collected at a 2048 × 2048-pixel resolution, and appropriate emission filters BA420-460, BA575IF, and BA510550 (Olympus, Tokyo, Japan) were used. Digital images were captured synchronously by two digital CMOS cameras (ORCA-Flash 4.0 V3; Hamamatsu, Hamamatsu City, Japan). Data collection was done with the capturing software cellSens (Olympus, Tokyo, Japan). For quantitative analysis of digital images, the ImageJ software (NIH, Bethesda, MD, USA) was used. The open-source software Icy (https://icy.bioimageanalysis.org)^35^ was used for 3D reconstruction and 4D visualization.

### Mitochondrial Membrane Potential Assessment

We utilized the tetraethylbenzimidazolylcarbocyanine iodide-based (JC-1) fluorescent probe and confocal microscopy to measure mitochondrial membrane potential in cells grown under MC and CS conditions. Mitochondrial membrane potential changes were analyzed using a previously described methodology.^43,44^ Samples were stained with 1 μM JC-1 fluorescent probe and incubated for 30 min at 37 °C. JC-1 selectively enters mitochondria and, in healthy cells with high mitochondrial membrane potential, forms complexes (J-aggregates) with intense red fluorescence. Upon mitochondrial damage (in apoptotic or unhealthy cells) and subsequent decrease of mitochondrial membrane potential, JC-1 remains in the monomeric form, which shows only green fluorescence. The ratio of green-to-red fluorescence is dependent specifically on the membrane potential but not on other factors.^47,48^ Following staining, cells were analyzed using the spinning disk confocal system IXplore SpinSR (Olympus, Tokyo, Japan). The ImageJ software (NIH, Bethesda, MD, USA) was used for image processing and quantification. To verify functional changes associated with either MC or CS conditions, cells were subjected to different depolarizing agents’ treatment: ionomycin (ION), 10 min 1 μM; carbonyl cyanide *m*-chlorophenylhydrazone (CCCP), 30 min 10 μM; and potassium cyanide (KCN), 5 mM, 30 min.

### Detection of Mitochondrial Reactive Oxygen Species (ROS)

We assessed mitochondrium-specific ROS utilizing quantitative confocal fluorescent imaging described previously.^49^ Briefly, cells were grown either in the monolayer culture (MC) or in collagen scaffolds (CSs). Afterward, cells were loaded with 0.5 μM MitoTracker red CM-H_2_XRos (Thermo Fisher Scientific, Waltham, MA, USA) for 15 min at 37 °C in the dark and imaged using the spinning disk confocal system IXplore SpinSR (Olympus, Tokyo, Japan). Quantitative analysis of MitoTracker red CM-H_2_XRos fluorescence imaged by the ImageJ software (NIH, Bethesda, MD, USA). In total, we analyzed *n* = 18–34 randomly selected fields per condition out of three independent experiments to achieve reliable statistical sampling. We used this methodology for mitochondrial ROS detection, not occasionally. Such quantitative confocal fluorescent imaging was shown to be a reliable measure for mitochondrial ROS.^49,50^ Previously, it was found that confocal fluorescent imaging assessment of mitochondrial ROS is in good agreement with electron spin resonance spectroscopy.^49^ Moreover, MitoTracker red CM-H_2_XRos was shown to have high mitochondrial specificity and high sensitivity for mitochondrial ROS detection.^49^ We intentionally avoided methods for mitochondrial ROS detection that are dependent on cell detachment like flow cytometry. It has been shown that cancer cells’ detachment from the extracellular matrix elevates ROS levels.^51^ Therefore, to minimize the influence of handling on the cellular production of ROS, we used minimally invasive quantitative confocal fluorescent imaging.^49^

### Fluorescent Image Processing and Quantification

Original microscopy images were acquired using the software cellSens (Olympus, Tokyo, Japan). For image processing and quantitative analysis, we used the ImageJ software (NIH, Bethesda, MD, USA). The open-source software Icy (https://icy.bioimageanalysis.org)^35^ was used for 3D reconstruction and 4D visualization.

To measure the length of cytoskeletal filaments F-actin and β-tubulin, we used the ImageJ plugin AnalyzeSkeleton.^52,53^ Scattered plots were created using SigmaPlot 13 (Systat Software, Palo Alto, CA, USA).

To perform effective and semiautomatized quantification of JC-1 green-to-red fluorescence ratio, we created automatization macros by recording them using the “Recorder” function in the ImageJ software (NIH, Bethesda, MD, USA). Contrast and brightness enhancement was performed using Macro 1 (supplemental macro material). Afterward, individual cells were selected using the ROI Manager function manually. Further, integrated densities for defined ROIs of red and green channels were measured, and then the ratio of integrated density for the red channel and integrated density for the green channel was calculated by Macro 2 (supplemental macro material). Digital images were presented as a multistack montage using Macro 3 (supplemental macro material). Finally, after manual selection of ROI for presentation, a montage for green, red, and merged channels was done using Macro 4 (supplemental macro material).

The LIVE/DEAD Viability/Cytotoxicity Kit was used to determine the cytotoxicity of high-fluence, low-power red (649 nm) laser light. This two-color fluorescence cell viability assay is based on the ability of calcein-AM to be retained within live cells, inducing an intense uniform green fluorescence and EthD-1 to bind the nuclei of damaged cells, thus producing a bright red fluorescence in dead cells.^54^ Cells were loaded with fluorescent probes calcein-AM (1 μM) and EthD-1 (4 μM) for 30 min. After labeling, cells were exposed to HFLP laser irradiation. Subsequently, images were captured using the high-resolution spinning disk confocal system IXplore SpinSR (Olympus, Tokyo, Japan). The fluorescence intensity of both dyes was measured at the respective time points and was normalized to the total fluorescence 30 min after dye loading utilizing the ImageJ software (NIH, Bethesda, MD, USA). To confirm the validity of the live/dead staining, cells were also treated with 20% ethanol for 10 min and subsequent imaging (data not shown).

Mitochondrial morphology was assessed utilizing a previously published methodology of image quantification.^55−57^ Mitochondrial morphology was depicted as fragmented (>75% of the mitochondria visible in the cell were small and round), intermediate (mixture of round and shorter tabulated), and tubulated (>75% of the mitochondria visible in the cell were long; higher interconnectivity). More than 100 cells/condition were randomly chosen and classified as described, and the percentage of each category was calculated.

### Human Liver Samples

Neoplastic liver tissues were obtained from 12 consecutive patients at the time of surgical resection performed at the Transplant Surgery Department of the Institute for Clinical and Experimental Medicine.^58^ Collection of clinical samples was accomplished in concordance with the standards of and approved by the Institute for Clinical and Experimental Medicine and Thomayer University Hospital Research Ethics Committee; all study subjects signed the informed consent document. The processing of samples was described in full detail previously.^58^

### Statistical Analysis

Kruskal–Wallis one-way analysis of variance on ranks with subsequent Newman–Keuls or Dunn’s test was used to evaluate the statistical significance of differences between multiple groups. For two-group comparisons, we utilized the Mann–Whitney *U* test. The MaxStat Pro 3.6 software (MaxStat Software, Cleverns, Germany) and SigmaPlot 13 (Systat Software, Palo Alto, CA, USA) were used to perform all statistical analyses. Differences were considered statistically significant at (*) *P* < 0.05.

Quantitative analysis of confocal microscopy images was done in accordance with published good practice guidelines.^59,60^ At least three independent experiments were performed for quantitative microscopy analysis. Each microscopy experiment included at least 10 randomly selected fields from each sample. We determined the sample size for each microscopy experiment according to previously published guidelines.^61^ Considering 95% confidence level and 0.9 statistical power, the sample size equals 30. Thus, we assessed at least 30 randomly selected cells for statistically relevant fluorescence microscopy image quantification.

Overall, we used sample determination for each experiment using guidelines published previously in ref (61) assuming 95% confidence level and 0.9 statistical power.

## Results and Discussion

### Characterization of 3D Collagen Scaffolds

**Figure 2:**
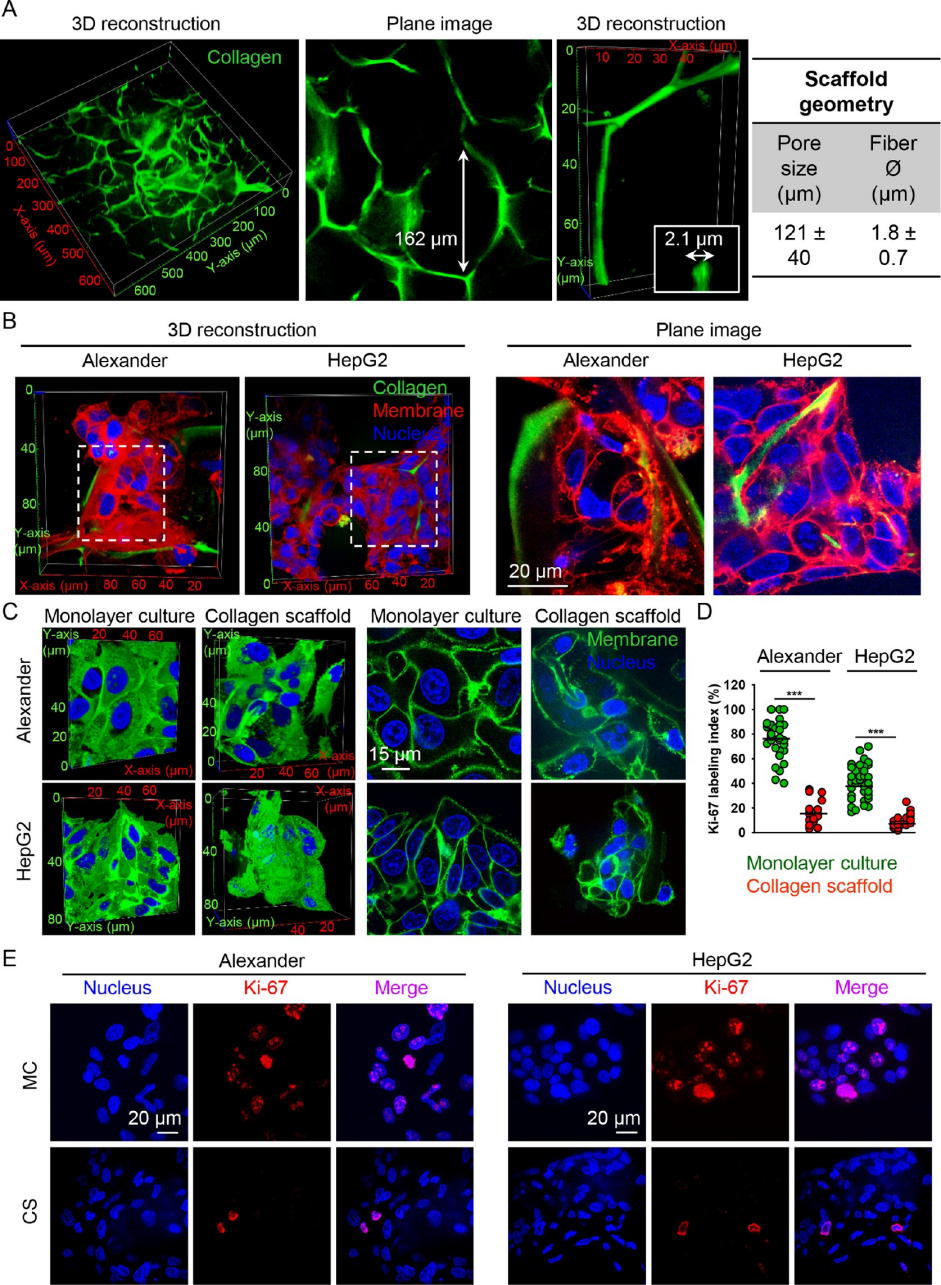
High-resolution confocal imaging of the collagen microstructure of collagen scaffolds (CSs). (A) ColF (green) fluorescent probe labels specifically collagen fibers. 3D reconstruction and plane images of collagen scaffold morphology as captured by confocal microscopy. Insert visualizes the fiber cross-section. 3D reconstruction was done using the open-source software Icy (https://icy.bioimageanalysis.org). Fiber diameter (*n* = 72 fibers) and pore size (*n* = 204 pores) were measured using the ImageJ software (NIH). Quantification of three independent experiments. (B) HepG2 and Alexander cells were grown in collagen scaffolds for 7 days. Cell membranes were labeled with CellMask Orange (red). Hoechst 33342 (blue) dye was used to counterstain nuclei. Collagen fibers were stained with ColF (green). Labeled cells were then imaged by high-resolution spinning disk confocal microscopy. 3D reconstruction was done using the open-source software Icy (https://icy.bioimageanalysis.org). Plane confocal images of the selected region are shown on left panels. (C) Morphological changes upon mechanical constraint of cells grown in the 3D-engineered microenvironment. HepG2 and Alexander cells were grown either in a standard monolayer culture (MC) or in collagen scaffolds (CSs). Cell membranes were labeled with CellMask Green (green). Hoechst 33342 (blue) dye was used to counterstain nuclei. Labeled cells were then imaged by confocal microscopy. 3D reconstruction was done using the open-source software Icy (https://icy.bioimageanalysis.org). Plane images were processed using the ImageJ software (NIH). (D) Cell proliferation dynamics was monitored using the anti-Ki67 antibody. Immunofluorescence confocal microscopy analysis of Ki-67 labeled Alexander and HepG2 cells that were cultivated either in MC or in CS and then fixed, permeabilized, and stained for Ki-67. Stained cells were imaged by confocal microscopy, and digital images were processed and quantified in ImageJ. The Ki-67 labeling index was determined. *n* = 20–39 randomly selected images out of four independent experiments. (***) *P* < 0.001 denotes significant differences with respect to the monolayer culture. (E) Examples of confocal microscopy imaging of Ki-67 staining. HepG2 and Alexander cells were cultivated and processed as in panel D. Staining for Ki-67 is presented in red. Hoechst 33342 (blue) dye was used to counterstain nuclei. Labeled cells were then imaged by confocal microscopy, and the images were processed using the ImageJ software (NIH).

We decided to analyze the interplay between mechanical cues and liver tumor cell metabolism in a soft 3D system in which mechanical constraint results in established slowing down of tumor cell proliferation (Figure 1).^33^ To experimentally modulate the extracellular mechanics of HepG2 and Alexander cells (Figure 1), we grew them on a standard glass-bottom dish (Cellvis, Mountain View, CA, USA) that served as the monolayer culture (MC) and soft elastic porous 3D collagen scaffolds (CSs) with storage modulus (*G*′) ∼94 Pa and loss modulus (*G*″) ∼0 Pa.^33^ We used a previously published cryostructuring technique to generate porous collagen scaffolds.^33^ It is known that the accumulation of collagen types I and III is associated with liver ECM reorganization during the course of various pathologies, including liver cancer.^8,62,63^ Thus, to have a more realistic 3D biomimetic microenvironment, we utilized collagen scaffolds in this study. We previously thoroughly characterized collagen scaffolds and determined their structure, porosity, diffusivity, and viscoelastic properties.^33^ Disc-shaped collagen scaffolds (∼5 × 5 × 3 mm) were characterized for pore size and fiber diameter using confocal microscopy. Indeed, it has been shown that sample preparation for scanning electron microscopy, which requires the dehydration of the scaffold, may strongly alter the 3D scaffold architecture, changing the size and shape of collagen fibers.^64^ Therefore, to visualize the 3D microstructure of the scaffold in a liquid environment, we labeled collagen fibers with the low-molecular-weight fluorescent probe Col-F (Figure 2A). Using the high-resolution and ultrafast confocal imaging system, we determined the average pore size of CS to be 121 ± 40 μm and the fiber diameter to be about 1.8 ± 0.7 μm (Figure 2A).

It is worth noting here that the precise 3D structure of the ECM enriching liver tumor microenvironment *in vivo* is not well described.^65,66^ Specifically, structural characteristics of collagen in the liver are not fully determined. Studies estimate the diameter of collagen fiber to be about 200–1000 nm.^66−68^ Samples of ECM from cirrhotic human livers were found to have pores of approximate size ∼150 μm.^66,69^

### Soft Microenvironment Changes the Morphology and Decreases the Proliferation of Liver Tumor Cells

We previously showed that HepG2 and Alexander cell culturing in 3D scaffolds slows down their proliferation without affecting viability.^33^ Additionally, we found that mechanically driven effects on cellular function are the most profound on the seventh day of culturing.^33^ Therefore, we selected this time point for further research. Indeed, both cell lines effectively populated the 3D environment of the collagen scaffold after 7 days (Figure 2B). Such growth of cells was accompanied by dramatic changes in the cellular shape compared to MC conditions (Figure 2C and Figure S1). In line with our previous results,^33^ HepG2 and Alexander cells became smaller in size and dramatically changed their shape (Figure 2C). Indeed, it became evident that external mechanical forces regulate the cellular shape.^70^ However, studies analyzing the mechanical regulation of HCC cellular behavior and functions rely on 2D substrates with modulated stiffness.^5,6,17,20,71,72^ Such models do not account for the additional mechanical cue that arises from 3D culturing itself: pressure of neighboring cells.^33^ We previously showed that Alexander and HepG2 cells experience a distribution of mechanical factors regulating the cellular shape and dormancy upon culturing in 3D CS.^33^ In line with our previous results,^33^ we revealed that culturing of both cell lines in soft 3D CS leads to a marked decrease of proliferation as evident by the lowering of the Ki-67 labeling index (Figure 2D,E). Of note, it was shown that dormant tumor cells express low levels of Ki-67.^73,74^ Interestingly, it was shown that Huh7 and HepG2 cells cultured on 2D soft (1 kPa stiffness) substrates undergo reversible cellular dormancy.^17^ In line with that research, we reveal that the soft 3D microenvironment can stimulate liver tumor cell dormancy by inhibiting the proliferation and lowering Ki-67 expression.

### Cytoskeletal Reorganization Modulated by the Stiffness of the Collagen Scaffold in Liver Tumor Cells

**Figure 3:**
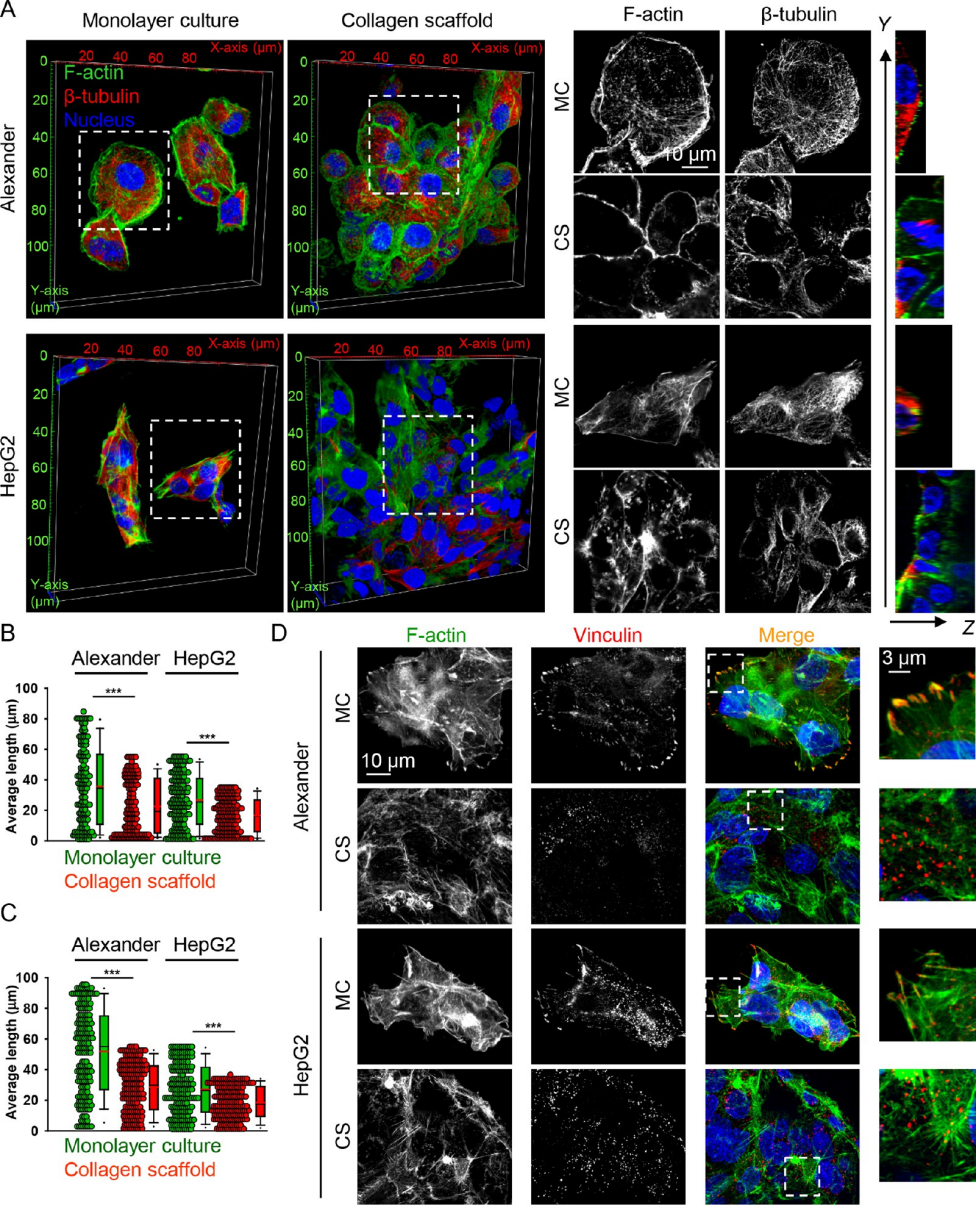
Cytoskeleton remodeling upon mechanical constraint of cells grown in the 3D-engineered microenvironment. (A) Alexander and HepG2 cells were grown for 7 days either in MC or CS conditions. F-actin (green) was stained using the ActinGreen 488 ReadyProbes reagent. β-Tubulin (red) was labeled using the anti-β-tubulin antibody. The nucleus (blue) was counterstained with Hoechst 33342. Labeled cells were imaged by high-resolution spinning disk confocal microscopy. 3D reconstruction was done using the open-source software Icy (https://icy.bioimageanalysis.org). Confocal plane images are shown for selected regions. (B) Length of F-actin filaments of cells grown in either MC (*n* = 150–197 cells) or CS (*n* = 159–199 cells) conditions. Quantification of three independent experiments. The length of F-actin filaments was determined using the ImageJ plugin AnalyzeSkeleton. (***) *P* < 0.001 denotes significant differences with respect to the monolayer culture. (C) Length of β-tubulin filaments of cells grown in either MC (*n* = 184–187 cells) or CS (*n* = 187 cells) conditions. Quantification of three independent experiments. The length of β-tubulin filaments was determined using the ImageJ plugin AnalyzeSkeleton. (***) *P* < 0.001 denotes significant differences with respect to the monolayer culture. (D) Focal adhesion analysis was performed using the anti-vinculin (red) antibody, and Hoechst 33342 was used as a counterstain for the nucleus (blue).

Generally, it is widely recognized that extrinsic mechanical forces propagate in cells through the cytoskeleton.^75^ Cytoskeletal filaments are coupled to the nuclear envelope, mitochondria, and other organelles, resulting in the transmission of physical constraints to cellular structures.^75^ Actin filaments and microtubules were shown to have an integral role in sensing the external stiffness of the microenvironment.^76−79^ Therefore, we tested how the soft microenvironment would reorganize cytoskeletal filaments. Confocal microscopy analysis of the cytoskeleton architecture revealed marked changes in the organization of actin filaments and microtubules (Figure 3A). To quantify these structural differences in the cytoskeleton, we utilized the ImageJ plugin AnalyzeSkeleton^52,53^ to measure the average filament length per selected cell. Alexander and HepG2 cell culturing in soft 3D scaffolds resulted in shorter, fragmented filaments of F-actin and β-tubulin (Figure 3A–C). These analyses confirmed a significant shift in actin filaments and microtubule organization of cells upon mechanical constraint. Further, analysis of vinculin distribution showed that in cells grown in soft 3D CS, vinculin was spot-like randomly distributed, whereas in stiff MC conditions, vinculin was allocated with F-actin fibers at the cellular border (Figure 3D). Vinculin is known to be concentrated in focal adhesion sites and reflects the adhesion of cells.^80^ Thus, taken together, our data indicated that in soft 3D CS, cells experience less adhesion and distinct mechanical cues compared to stiff MC conditions.

### Soft 3D Microenvironment Promotes Fission and Depolarizes Mitochondria

**Figure 4:**
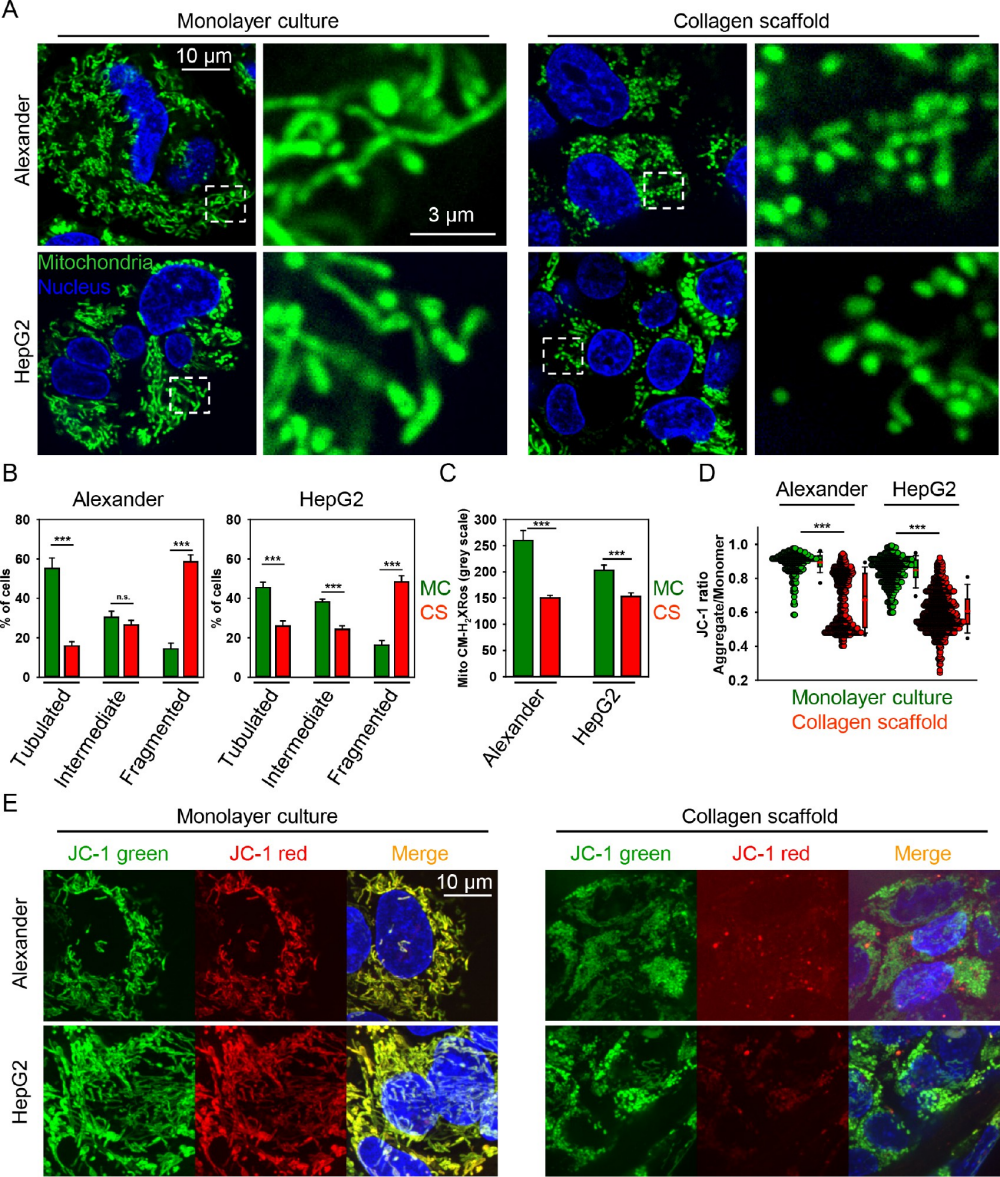
Mitochondrial dynamics is mechanically modulated. (A) Alexander and HepG2 cells were cultivated for 7 days in either MC or CS conditions. Mitochondria (green) were labeled using MitoTracker Green. Hoechst was used for the nucleus (blue) as a counterstain. Labeled cells were imaged by confocal microscopy, and the digital images were processed using the ImageJ software (NIH). (B) Quantitative analysis of mitochondrial morphologies. The percentage of cells with indicated mitochondrial morphologies was determined as a percentage of the total number of cells counted. Quantification of three independent experiments. The data are presented as mean ± SE; *n* ≥ 100 cells per experiment. (***) *P* < 0.001 denotes significant differences with respect to the monolayer culture. (C) Mitochondria-specific reactive oxygen species were measured in Alexander and HepG2 cells cultivated for 7 days in either MC or CS conditions. Cells were labeled with the fluorescent probe Mito CM-H_2_XRos. Labeled cells were imaged by confocal microscopy, and the digital images were quantified using the ImageJ software (NIH). *n* = 18–34 randomly selected images out of three independent experiments. (***) *P* < 0.001 denotes significant differences with respect to the monolayer culture. (D) Alexander and HepG2 cells were cultivated for 7 days in either MC or CS conditions. Mitochondrial membrane potential (ΔΨm) was measured using the JC-1 fluorescent probe. Labeled cells were imaged by confocal microscopy, and the digital images were quantified using the ImageJ software (NIH). JC-1 ratio of cells grown in either MC (*n* = 257–421 cells) or CS (*n* = 397–735 cells) conditions. Quantification of three independent experiments. (***) *P* < 0.001 denotes significant differences with respect to the monolayer culture. (E) Example of mitochondrial membrane potential (ΔΨm) assessment in Alexander and HepG2 cells cultivated for 7 days in either MC or CS conditions. Cells were labeled using the JC-1 fluorescent probe. Hoechst 33342 was used as a counterstain for the nucleus (blue). Labeled cells were imaged by confocal microscopy, and the digital images were processed using the ImageJ software (NIH). Green, red, and merged channels are assembled as multistack montage images with maximum intensity projection.

Emerging evidence highlights an important role of actin filaments and microtubules in controlling mitochondrial fission/fusion balance and function.^81−83^ Additionally, it has been showed that mechanical forces may directly modulate mitochondrial dynamics and metabolism.^75,84^ Therefore, we next assessed how soft 3D CS would affect mitochondrial activity and dynamics. Indeed, mitochondria in cells grown in stiff MC conditions showed highly networked and tubular shapes (Figure 4A and Figure S2). In contrast, cells grown in soft 3D CS displayed an increased number of fragmented and granular mitochondria (Figure 4A and Figure S2). Quantitatively, culturing of both cell lines in soft 3D CS resulted in a sharp increase in the percentage of cells with fragmented mitochondria and a concomitant decrease in the percentage of cells with tubulated mitochondria (Figure 4B).

**Figure 5:**
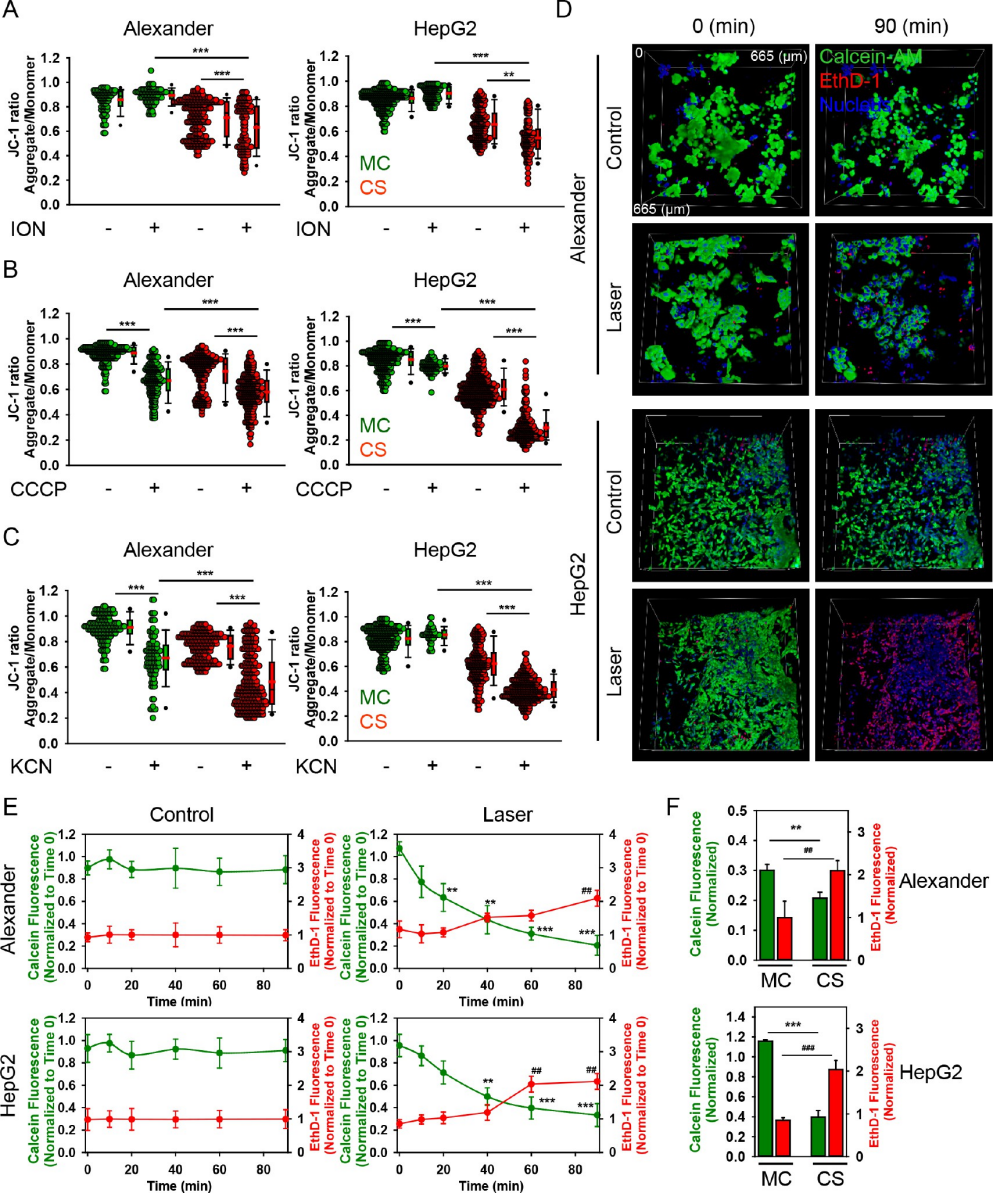
Mechanical constraint leads to mitochondrial depolarization in cells grown in the 3D-engineered microenvironment. Comparative analysis of mitochondrial membrane potential sensitivity of cells grown in either MC or CS conditions to different depolarizing agents’ treatment: (A) ionomycin (ION), 10 min 1 μM; (B) carbonyl cyanide *m*-chlorophenylhydrazone (CCCP), 30 min 10 μM; and (C) potassium cyanide (KCN), 5 mM, 30 min. Alexander and HepG2 cells were cultivated for 7 days in either MC or CS conditions, stained with 1 μM JC-1, and treated with different depolarizing agents. Mitochondrial membrane potential (ΔΨm) was analyzed by confocal microscopy. The JC-1 ratio was calculated using the ImageJ software (NIH). Quantification of three independent experiments; *n* ≥ 100 cells per experiment. (**) *P* < 0.01 and (***) *P* < 0.001 denote significant differences. (D) Both cell lines (HepG2 and Alexander) were cultivated in CS for 7 days. Then, cells were labeled with the LIVE/DEAD Viability/Cytotoxicity Kit. Specifically, cells were loaded with calcein-AM (green) and ethidium homodimer (EthD-1, red). Hoechst 33342 was used as a counterstain for the nucleus (blue). Labeled cells were subjected to 649 nm high-fluence. low-power (HFLP) laser irradiation for 90 min. Control cells were untreated. Images were acquired by confocal microscopy. 3D reconstruction was done using the open-source software Icy (https://icy.bioimageanalysis.org). (E) Kinetics of cell dying upon high-fluence, low-power (HFLP) laser irradiation. Alexander and HepG2 cells were cultivated and stained as described in panel D. The ImageJ software (NIH) was used for image processing and quantification. The fluorescence intensity of both dyes was measured at the respective time points and was normalized to total fluorescence. Data are expressed as means ± SEM (*n* = 3, three independent experiments); *t* = 0 time point served as control. (^##^) *P* < 0.01, (**) *P* < 0.01, and (***) *P* < 0.001 denote significant differences. (F) Alexander and HepG2 cells were cultivated for 7 days in either MC or CS conditions, and then cells were loaded with calcein-AM (green) and ethidium homodimer (EthD-1, red). Labeled cells were subjected to 649 nm high-fluence, low-power (HFLP) laser irradiation for 90 min and imaged by confocal microscopy. The fluorescence intensity of both dyes was measured using the ImageJ software (NIH). Data are expressed as means ± SEM (*n* = 3, three independent experiments). (^##^) *P* < 0.01, (^###^) *P* < 0.001, (**) *P* < 0.01, and (***) *P* < 0.001 denote significant differences.

Mitochondrial reactive oxygen species (mitoROS) are known byproducts of normal mitochondrial metabolism and homeostasis, mainly produced during the oxidative phosphorylation process.^85^ Production of mitoROS is tightly associated with mitochondrial fission.^85^ Thus, we analyzed mitoROS production utilizing a specific fluorescent probe. We revealed that cells cultured in MC conditions produced significantly more mitoROS than cells grown in soft 3D CSs (Figure 4C). These data tentatively proposed that the soft microenvironment decreased the oxygenic metabolism of mitochondria. It is worth noting that accumulation of fragmented mitochondria was linked to decreased mitochondrial membrane potential and energetics.^86,87^ Furthermore, cells with elevated mitochondrial fission showed poor proliferation and decreased cellular respiration.^86^ Therefore, we next assessed whether mechanical regulation of mitochondrial fragmentation was associated with mitochondrial membrane potential (ΔΨm) changes. In fact, we revealed that ΔΨm was significantly higher for cells grown under stiff MC conditions in comparison with cells cultured on soft 3D CS (Figure 4D,E). To further validate the mechanical regulation of ΔΨm, we challenged Alexander and HepG2 cells cultured in either stiff MC or soft 3D CS conditions with chemically distinct ΔΨm depolarizing agents (i.e., ionomycin,, carbonyl cyanide *m*-chlorophenylhydrazone, and potassium cyanide). Both cell lines grown on soft 3D CS showed ΔΨm to be dramatically susceptible to all depolarizing agents in comparison with cells cultured under MC conditions (Figure 5A–C). Taken together, these data indicate that the soft 3D microenvironment promotes ΔΨm depolarization.

**Figure 6:**
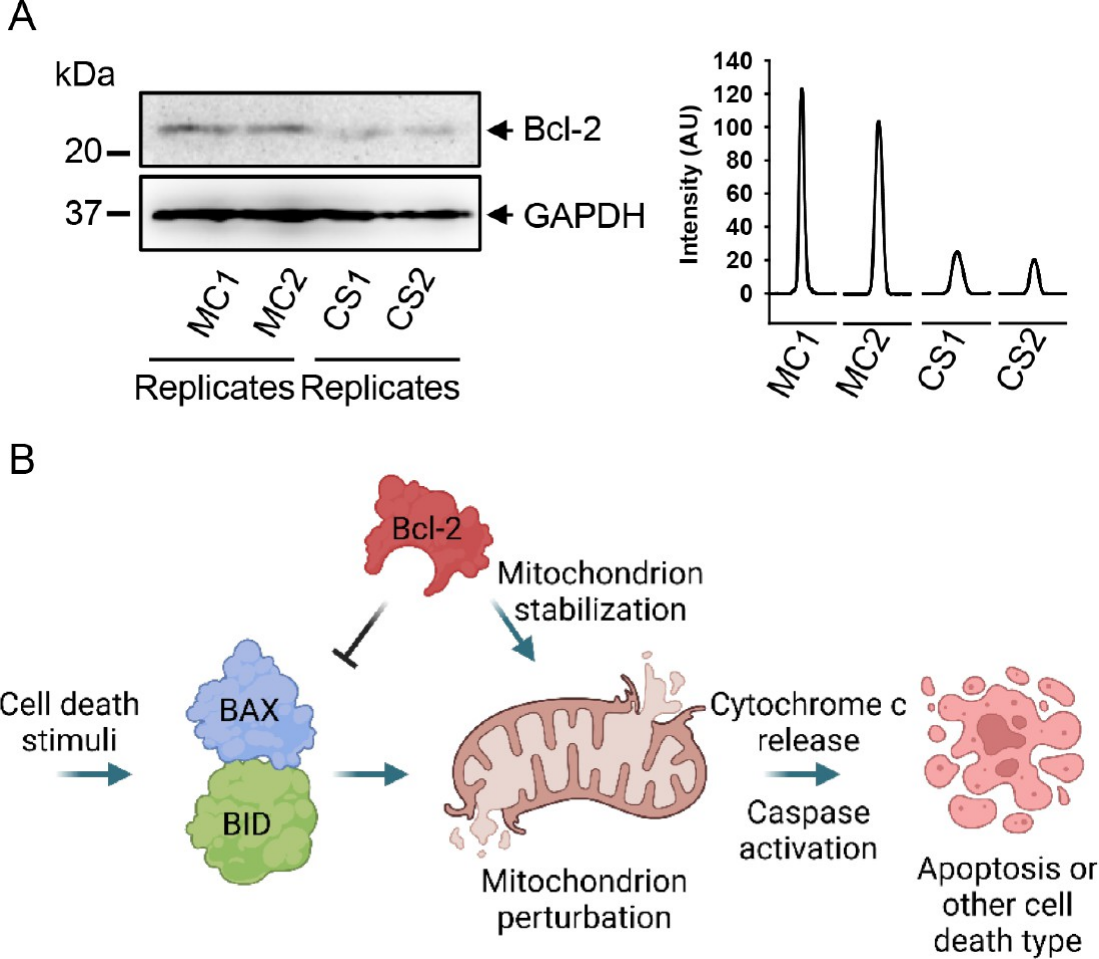
Mechanical constraint downregulates the expression of Bcl-2 protein in HepG2 cells. (A) HepG2 cells were cultivated for 7 days in either MC or CS conditions. Bcl-2 expression was analyzed in whole cell lysates of HepG2 cells by immunoblotting; GAPDH—control of equal protein loading. The graph shows the densitometric quantification of Bcl-2 immunoblots. (B) Scheme that shows the prosurvival Bcl-2 protein. Activation of the proapoptotic effectors Bcl-2-like protein 4 (BAX) and Bcl-2 homologous antagonist/killer (BAK) protein leads to the disruption of the mitochondrial membrane. Mitochondrial membrane perturbation results in the release of cytochrome *c* and promotes caspase activation. Taken together, those events trigger cell death. Bcl-2 protein contracts cell death execution by inhibiting BAX and BAK proteins and/or stabilizing the mitochondrial membrane. Created with BioRender.com.

Next, to link mechanically modulated ΔΨm depolarization with functional consequences, we analyzed cell survival under high-fluence, low-power (HFLP) laser irradiation. We and others have previously showed that HFLP laser irradiation in the red (620–760 nm) region results in cell death via marked ΔΨm depolarization.^43,44,88^ In fact, HFLP 649 nm laser irradiation significantly decreased the viability of HepG2 and Alexander cells cultured on soft 3D CS (Figure 5D,E and Movies S1–S4). Interestingly, we previously showed that HepG2 cells cultured in MC conditions were insensitive to HFLP 649 nm laser irradiation as a result of elevated levels of the antiapoptotic protein Bcl-2.^44^ Indeed, comparative analysis of cells cultured in either stiff MC or soft 3D CS conditions revealed that cell growth in 3D CS conditions makes both cell lines more susceptible to HFLP laser-induced toxicity (Figure 5F). These data confirmed our observations revealing that the soft 3D microenvironment promotes marked ΔΨm depolarization, making cells more sensitive to various depolarizing treatments. To validate the involvement of Bcl-2 in HepG2 sensitivity toward laser-induced cytotoxicity, we assessed protein expression levels of this protein in cells cultured in either stiff MC or soft 3D CS conditions. Immunoblot analysis revealed that culturing HepG2 cells in soft 3D CS conditions leads to a marked decrease of Bcl-2 expression level (Figure 6A). Of note, generally, proteins of the Bcl-2 family play a crucial role in the global regulation of mitochondrial homeostasis and determine the commitment of cells to apoptosis.^89,90^ Prosurvival proteins, such as Bcl-2 itself, either compete with proapoptotic proteins (e.g., BAK, BAX) or block mitochondrial dysfunction by modulating proton flux (Figure 6B).^90^ Thus, Bcl-2 prevents apoptotic signaling. Consequently, it was shown that elevated levels of Bcl-2 make cells resistant to different stimuli triggering apoptosis via dysregulation of mitochondrial function.^38,90,91^ Therefore, we conclude that downregulation of Bcl-2 expression upon culturing HepG2 cells in soft 3D CS conditions results in the sensitization of cells toward HFLP laser-induced toxicity.

To summarize, liver tumor cell culturing in the soft 3D microenvironment results in the low production of mitoROS, ΔΨm depolarization, and elevated mitochondrial depolarization-sensitive cytotoxicity. These data taken together represent an indicator of reduced mitochondrial function of cells grown in soft 3D CS.

### Soft Microenvironment Elevates Glycolysis in Liver Tumor Cells

**Figure 7:**
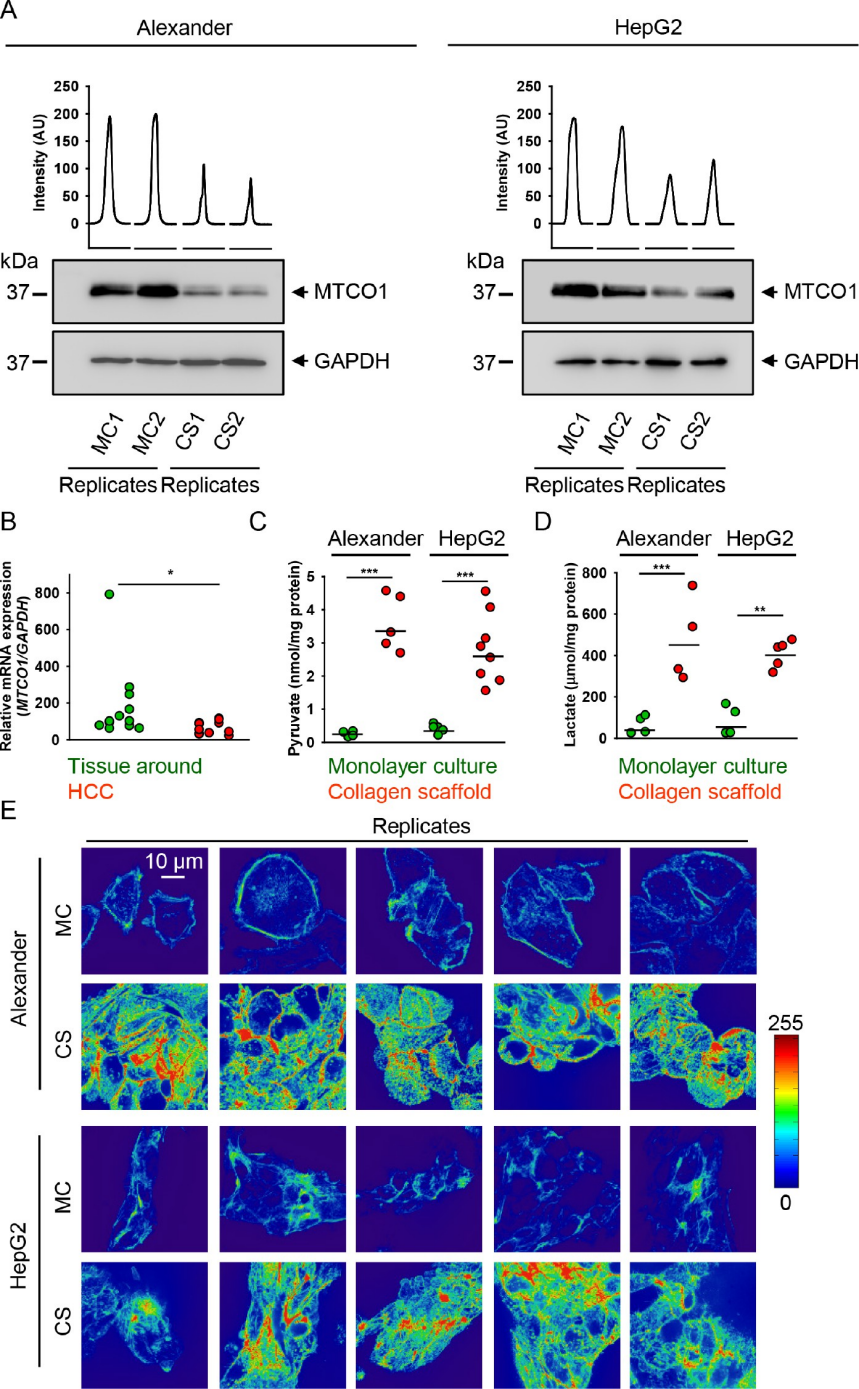
Mechanical constraint modulates glycolysis in human liver tumor cells. (A) Alexander and HepG2 cells were cultivated for 7 days in either MC or CS conditions. Mitochondrially encoded cytochrome *c* oxidase I (MT-CO1) expression was analyzed in whole cell lysates of HepG2 and Alexander cells by immunoblotting; GAPDH—control of equal protein loading. The graphs show the densitometric quantification of MT-CO1 immunoblots. (B) *MT-CO1* gene expression level analysis in liver samples from hepatocellular carcinoma (HCC) patients. The mRNA levels of *MT-CO1* in 12 HCC tissues and paired adjacent normal tissues around were detected by qPCR. The relative gene expression was normalized to *GAPDH* expression and calculated using the 2^–ΔΔCT^ method. (*) *P* < 0.05 denotes significant (*P* = 0.0153) difference determined by the Mann–Whitney test. Assessment of intracellular pyruvate (C) and lactate (D) concentrations in Alexander and HepG2 cells cultivated in either MC or CS. Alexander and HepG2 cells were cultivated for 7 days in either MC or CS conditions, and then cell extracts were collected and deproteinized using <10 kDa MWCO filter spin. Subsequently, levels of pyruvate (C) and lactate (D) were determined using the pyruvate fluorescence assay or lactate fluorescence assay, respectively (both from Sigma Aldrich). *n* = 4–8 samples out of three independent experiments. (**) *P* < 0.01 and (***) *P* < 0.001 denote significant differences. (E) Confocal fluorescence analysis of the cytoskeleton organization of cells under mechanical constraint. Alexander and HepG2 cells were grown for 7 days in either MC or CS conditions. F-actin was stained using the ActinGreen 488 ReadyProbes reagent. F-actin tension was assessed by measuring fluorescence distribution and intensity utilizing confocal microscopy. The fluorescence distribution and intensity of F-actin are shown in the reported pseudo-color scale (0–255). Digital images were processed using the ImageJ software (NIH). F-actin tension was visualized using the look-up table *Physics* in the ImageJ software (NIH).

It is worth noting that reducing mitochondrial function by inhibiting oxidative phosphorylation is a general strategy of HCC cells to decrease total oxygen consumption, adapt to hypoxia, and stimulate cancer progression.^92,93^ HCC progression was shown to be associated with the downregulation of enzymes mediating oxidative phosphorylation, including cytochrome *c* oxidase.^94^ Interestingly, the human protein atlas links low mitochondrially encoded cytochrome *c* oxidase I (*MTCO1*) expression in liver HCC with a lower survival rate.^95^ Thus, we analyzed whether the expression of *MTCO1* is affected by mechanical cues. Indeed, the protein expression level of MTCO1 was significantly lower in cells grown in soft 3D CS in comparison with stiff MC conditions as evident by immunoblot analysis (Figure 7A). Importantly, there were no concomitant changes in the expression of any genes related to drug metabolism enzymes (Table S5) or inflammation-related genes (Table S6). Further, to have more support for our findings, we analyzed *MTCO1* gene expression in HCC tissues from an unselected cohort of 12 HCC specimens obtained at the time of tumor resection or biopsy. Indeed, *MTCO1* expression was lower in tumors compared to adjacent tissues (Figure 7B). This result supports previous data on cell cultures given the fact that edges of HCC tend to be stiffer in comparison with the core of the tumor.^96^ These data taken together indicate that culturing of liver tumor cells in soft 3D CS leads to specific metabolic rewiring of cells toward lowering mitochondria activity.

It is worth noting here that although mechanical stimuli of the cellular surrounding microenvironment have a strong effect on controlling cellular function, other physical cues (viscosity, geometry, and nanotopography) show a strong role in modulating cellular responses.^97,98^ Those different physical cues are interconnected. For example, the nanotopography of ECM affects the protein adsorption, which in turn dramatically modulates the conformation and distribution density of ECM, leading to changes in cellular responses.^99^ Additionally, physical cues originating from the microenvironment strongly modulate cell and tissue function and behavior during progression of many diseases.^97^ Also, the pathological development of many diseases is associated with changes of values in stiffness, viscosity, curvature, and ECM architecture.^97^ For instance, cancer progression has been linked with elevated stiffness, increased viscosity of extracellular fluid, and changes in geometrical constraint parameters of fibrillar ECM proteins; for review, see ref (97) and references therein. Nanotopography has been shown to regulate cell function through nuclear deformation.^100^ In fact, nuclear deformation induced by nanotopography modulated cell proliferation and type I collagen production.^100^ Furthermore, the nanotopography of the microenvironment may stimulate directed migration (i.e., topotaxis) of cancer cells.^101^ An additional very important physical factor of ECM that regulates cellular behavior is geometrical confinement of cells.^102,103^ Geometric constraint originating from ECM plays a crucial role in the modulation of different cellular functions.^102−105^ In fact, cell geometry is tightly interlinked with the regulation of nuclear shape, cytoskeleton reorganization, chromatin compaction, gene expression, growth, apoptosis, and cell division.^102,103,106,107^ Thus, confinement, stiffness, and topology of the microenvironment are crucial physical factors that modulate various cellular responses.^105^

Recent studies indicate that cellular metabolic activity might be regulated by external mechanical cues.^34,75^ Specifically, normal human bronchial epithelial cells transferred from stiff to soft 2D substrates showed decreased glycolysis rates.^34^ Epithelial lung cancer cells were found to be nonsensitive to glycolysis regulation by environmental mechanical changes having stabilized glycolysis.^34^ However, there is emerging evidence that cancer cells possess a heterogeneous and flexible metabolic profile.^108^ Such heterogeneity can be modulated by various factors, including interactions with ECM.^109^ Taking into account the morphologic intratumoral heterogeneity^21^ and mechanical heterogeneity of tumors,^22,23^ we hypothesized that tumor cell metabolism might be mechanically affected by mechanical cues that arise from the surrounding 3D microenvironment. Indeed, in both cell lines cultured in soft 3D CS, lactate and pyruvate concentrations were increased relative to stiff MC controls (Figure 7C,D). The accumulation of both lactate and pyruvate strongly suggested an increased glycolysis upon the soft mechanical surrounding of liver tumor cells. It has been shown that F-actin stress-fiber formation spatially protects from proteasomal degradation of phosphofructokinase (the rate-limiting metabolic enzyme), resulting in an elevated rate of glycolysis.^34^ Under relaxing mechanical cues, phosphofructokinase degradation is upregulated by PFK-targeting E3 ubiquitin ligase tripartite motif (TRIM)-containing protein 21 released from stress fibers.^34^ In fact, it was observed that TRIM21 strongly colocalizes with F-actin fibers.^34^ Importantly, when stress-fiber formation was increased, there was a concomitant elevation of TRIM21 localization on F-actin.^34^ Of note, it has been shown that TRIM21 targets the phosphofructokinase 1 platelet isoform (PFKP), leading to its polyubiquitylation and degradation.^110^ Moreover, TRIM21 knockdown results in enhanced PFKP expression.^34^ Therefore, the F-actin architecture regulates PFKP degradation by sequestration of TRIM21.^34^ We found that F-actin fiber reorganization in soft 3D CS results in higher intracellular fiber tension in comparison with stiff MC conditions (Figure 7E). The F-actin filament density proportionally grows as the color changes from blue to red, indicating an increase in fiber tension (Figure 7E).

## Conclusions

In this work, we performed the design and synthesis of soft 3D collagen scaffolds that replicate a pathological mechanical environment of liver tumors. Utilizing those scaffolds, we evaluated the functional activity of liver tumor cells subjected to mechanical cues. We intended to verify the hypothetical link between mechanically modulated cell proliferation and metabolic rewiring of liver tumor cells. We found that culturing of HepG2 and Alexander cells in soft 3D CS leads to the upregulation of glycolysis. We revealed that the soft 3D CS microenvironment results in reducing mitochondrial function. The soft 3D microenvironment increases fission and leads to marked depolarization of mitochondria. Changes in mitochondria activity were found to be associated with the downregulation of MTCO1 protein expression in the soft 3D CS microenvironment. We suggest that the soft microenvironment induces liver tumor cellular dormancy accompanied by reduced mitochondrial activity and elevated glycolysis.

We understand that there are certain limitations in our study. The liver ECM in healthy tissues and cancer pathology is more complex and is composed of collagen types I, III, IV, and V; fibronectin; elastin; and many other proteins.^111,112^ The holistic presentation of ECM is depicted by introducing the term “matrisome”, e.g., combination of molecules constituting ECM, matrix-associated enzymes and their inhibitors, growth factors, and the receptors for ECM.^98^ The liver ECM is a very complex and heterogeneous entity that is so far difficult to mimic utilizing current 3D cell culture models. A comprehensive landscape of physical parameters and mechanical properties of ECM of the liver is still not fully understood.^111,112^ Consequently, it means that understanding how mechanical cues originate from the 3D microenvironment is a formidable task. However, standardized 3D-engineered cell culture systems can help decipher the quantitative specifications of the mechanically driven modulation of cellular functions.

We believe that such soft 3D CS platforms can be very useful to study the basic foundations of mechanically regulated cellular dormancy and metabolic rewiring of various liver (potentially other tissues as well) tumor cells. Expanding the knowledge on mechanically regulated tumor metabolism would provide a basis for deciphering the pathophysiology of liver tumors and create a useful platform for the design of therapies targeting the mechanotransduction in cancer.

## Supporting Information Available

4D visualization of the viability of control (untreated) Alexander cells (Movie S1) (MP4)
4D visualization of the viability of laser-irradiated Alexander cells (Movie S2) (MP4)
4D visualization of the viability of control (untreated) HepG2 cells (Movie S3) (MP4)
4D visualization of the viability of laser-irradiated HepG2 cells (Movie S4) (MP4)
List of chemical probes used in the study (Table S1); list of fluorescent probes used in the study (Table S2); list of antibodies used in the study (Table S3); list of TaqMan probes used for qPCR (Table S4); quantitative PCR results for cytochrome gene expression in Alexander and HepG2 cells grown either in a standard monolayer culture (MC) or in collagen scaffolds (CSs) (Table S5); quantitative PCR results for selected inflammation-related genes’ expression in Alexander and HepG2 cells grown either in a standard monolayer culture (MC) or in collagen scaffolds (CSs) (Table S6); morphological changes upon mechanical constraint of cells grown in a 3D-engineered microenvironment (Figure S1); Alexander and HepG2 cells were cultivated for 7 days in either MC or CS conditions; mitochondria (green) were labeled using MitoTracker Green (Figure S2); characterization of the HFLP laser system (Figure S3); uncropped immunoblot scans; and ImageJ macro supplemental material (PDF)

## Supplementary Material

ab2c01518_si_001.avi

ab2c01518_si_002.avi

ab2c01518_si_003.avi

ab2c01518_si_004.avi
