# Supplementary material for: Mechanical Regulation of Mitochondrial Dynamics and Function in a 3D-Engineered Liver Tumor Microenvironment
Source: ACS Biomater Sci Eng. 2023 Mar 31;9(5):2408–25. doi: 10.1021/acsbiomaterials.2c01518 (PMC10170482; doi:10.1021/acsbiomaterials.2c01518)
Supplement: Supplementary file 5 — ab2c01518_si_005.pdf [file ab2c01518_si_005.pdf]

Supporting Information

Mechanical Regulation of Mitochondria Dynamics and Function in 3D-Engineered Liver Tumor Microenvironment

Adam Frtús [a], Barbora Smolková [a], Mariia Uzhytchak [a], Mariia Lunova [a,b], Milan Jirsa [b], Yuriy Petrenko [c], Alexandr Dejneka [a], and Oleg Lunov [a,]*

[a] Department of Optical and Biophysical Systems, Institute of Physics of the Czech Academy of Sciences, Prague, 18221, Czech Republic

[b] Institute for Clinical & Experimental Medicine (IKEM), Prague, 14021, Czech Republic

[c] Department of Neuroregeneration, Institute of Experimental Medicine of the Czech Academy of Sciences, Prague, 14220, Czech Republic

*Email: lunov@fzu.cz

Table S1. List of chemical probes used in the study.

Reagent	Manufacturer	Catalogue No
PBS	Gibco	10010015
Minimum Essential Medium Eagle	BioConcept	1-31S01-I
Glutamine	Serana Europe	RGL-001-100ML
Triton-X100	PanReac AppliChem	A4975,0100
Micro BCA Protein Assay Kit	Thermo Fisher Scientific	23235
RIPA buffer	Millipore	20188
Protease Inhibitor Cocktail	Sigma Aldrich	P8340-1ML
Phosphatase Inhibitor Cocktail 3	Sigma Aldrich	P0044-1ML
Paraformaldehyde	VWR	100503-917
BSA	Sigma Aldrich	2153
Blotting-Grade Blocker	Bio-Rad	170-6404
Clarity Max ECL Western Blotting Substrate	Bio-Rad	1705062
Collagen	VUP medical	011-0000
Acetic Acid	Sigma Aldrich	695092
N-(3-Dimethylaminopropyl)-N´-ethylcarbodiimide hydrochloride	Sigma Aldrich	E7750
Ethanol	Sigma Aldrich	1.07017
Fetal Bovine Serum	Gibco	10270-106
Penicilin/Streptomycin	Biosera	XC-A4122/100
Pyruvate Assay	Sigma Aldrich	MAK071
Lactate Assay	Sigma Aldrich	MAK064
Micro BCA Protein Assay Kit	Thermo Fisher Scientific	23235
RNeasy Mini Kit	Qiagen	74104
RNase-Free DNase Set	Qiagen	79254
Maxima H Minus First Strand cDNA Synthesis Kit	Thermo Fisher Scientific	K1652
Fast Advanced TaqMan Gene expression Master Mix	Thermo Fisher Scientific	4444557

Table S2. List of fluorescent probes used in the study.

Probe/Kit	Manufacturer	Catalogue No
Hoechst 33342	Thermo Fisher Scientific	62249
ColF	ImmunoChemistry Technologies	6346
ActinGreen™ 488 ReadyProbes™ Reagent	Thermo Fisher Scientific	R37110
Propidium Iodide	Thermo Fisher Scientific	R37108
CellMask green	Thermo Fisher Scientific	C37608
CellMask orange	Thermo Fisher Scientific	C10045
JC-1	Thermo Fisher Scientific	T3168
Mito CM-H_2XRos	Thermo Fisher Scientific	M7513
MitoTracker® Green	Thermo Fisher Scientific	M7514
LIVE/DEAD® Viability/Cytotoxicity Kit	Thermo Fisher Scientific	L3224

Table S3. List of antibodies used in the study.

Antibody	Clone/catalogue No	Dilution		Manufacturer
		WB	IF	
Anti- β-Tubulin	D2N5G/ 15115	N.A.	1:100	Cell Signaling Technology
Anti-Ki67	Ab15580	N.A.	1:1000	Abcam
Anti-GAPDH	ab226408	1:1000	N.A.	Abcam
Anti- mouse-HRP	1858413	1:10 000	N.A.	Pierce Biotechnology
Anti-rabbit- HRP	1858415	1:10 000	N.A.	Pierce Biotechnology
AlexaFluor 568 goat anti-rabbit IgG	A-11011	N.A.	1:100	Thermo Fisher Scientific
Anti-MTCO1	1D6E1A8/ab14705	1:1000	1:100	Abcam
Anti-Vinculin	hVIN-1/V 9131	N.A.	1:100	Sigma Aldrich
Anti-Bcl-2	15071	1:1000	N.A.	Cell Signaling Technology

N.A. – not applicable; WB – western blot; IF – immunofluorescence.

Table S4. List of TaqMan probes used for qPCR.

Name	Gene	Assay ID	Manufacturer

cytochrome P450 family 3 subfamily A member 4	*CYP3A4*	Hs00604506_m1	Thermo Fisher Scientific
cytochrome P450 family 2 subfamily B member 6	*CYP2B6*	Hs04183483_g1	Thermo Fisher Scientific
cytochrome P450 family 2 subfamily C member 8	*CYP2C8*	Hs00946140_g1	Thermo Fisher Scientific
cytochrome P450 family 2 subfamily E member 1	*CYP2E1*	Hs00559367_m1	Thermo Fisher Scientific
cytochrome P450 family 2 subfamily C member 19	*CYP2C19*	Hs00426380_m1	Thermo Fisher Scientific
C-X-C motif chemokine ligand 9	*CXCL9*	Hs00171065_m1	Thermo Fisher Scientific
interferon gamma	*IFNG*	Hs00174143_m1	Thermo Fisher Scientific
interferon alpha inducible protein 27	*IFI27*	Hs00271467_m1	Thermo Fisher Scientific
glyceraldehyde-3-phosphate dehydrogenase	*GAPDH*	Hs02786624_g1	Thermo Fisher Scientific
mitochondrially encoded cytochrome c oxidase I	*MT-CO1*	Hs02596864_g1	Thermo Fisher Scientific

Table S5. Quantitative PCR results for cytochromes genes expression in Alexander and HepG2 cells grown either in standard monolayer culture (MC) or in collagen scaffolds (CS).

**Gene**	**Expression value**							
	Alexander MC		Alexander CS		HepG2 MC		HepG2 CS	
	Average	SD	Average	SD	Average	SD	Average	SD
CYP3A4	3.147×10^{-6}	2.376×10^{-6}	4.808×10^{-6}	4.534×10^{-6}	5.072×10^{-5}	0.711×10^{-5}	3.389×10^{-5}	1.482×10^{-5}
CYP2B6	2.385×10^{-4}	0.190×10^{-4}	3.256×10^{-4}	0.709×10^{-4}	0.488×10^{-4}	0.087×10^{-4}	0.359×10^{-4}	0.079×10^{-4}
CYP2C8	0.404×10^{-4}	0.044×10^{-4}	0.224×10^{-4}	0.068×10^{-4}	0.185×10^{-4}	0.065×10^{-4}	0.092×10^{-4}	0.025×10^{-4}
CYP2E1	0.249×10^{-4}	0.032×10^{-4}	0.402×10^{-4}	0.049×10^{-4}	0.429×10^{-4}	0.115×10^{-4}	0.515×10^{-4}	0.088×10^{-4}

CYP2C19	0.064	0.215	0.002	0.001	1.078×10^{-5}	0.233×10^{-5}	0.401×10^{-5}	0.503×10^{-5}

SD – standard deviation; The relative gene expression was normalized to *GAPDH* expression and calculated using the $2^{-\Delta\Delta CT}$ method.

Table S6. Quantitative PCR results for selected inflammation related genes expression in Alexander and HepG2 cells grown either in standard monolayer culture (MC) or in collagen scaffolds (CS).

**Gene**	**Expression value**							
	Alexander MC		Alexander CS		HepG2 MC		HepG2 CS	
	Average	SD	Average	SD	Average	SD	Average	SD
CXCL9	0.001	0.003	0.005	0.010	0.0005	0.0007	0.001	0.003
IFNG	0.024×10^{-4}	0.005×10^{-4}	0.019×10^{-4}	0.001×10^{-4}	0.137×10^{-4}	0.242×10^{-4}	0.026×10^{-4}	0.004×10^{-4}
IFI27	2.822×10^{-4}	0.758×10^{-4}	1.297×10^{-4}	0.918×10^{-4}	0.863×10^{-4}	0.523×10^{-4}	0.631×10^{-4}	0.135×10^{-4}

SD – standard deviation; The relative gene expression was normalized to *GAPDH* expression and calculated using the $2^{-\Delta\Delta CT}$ method.

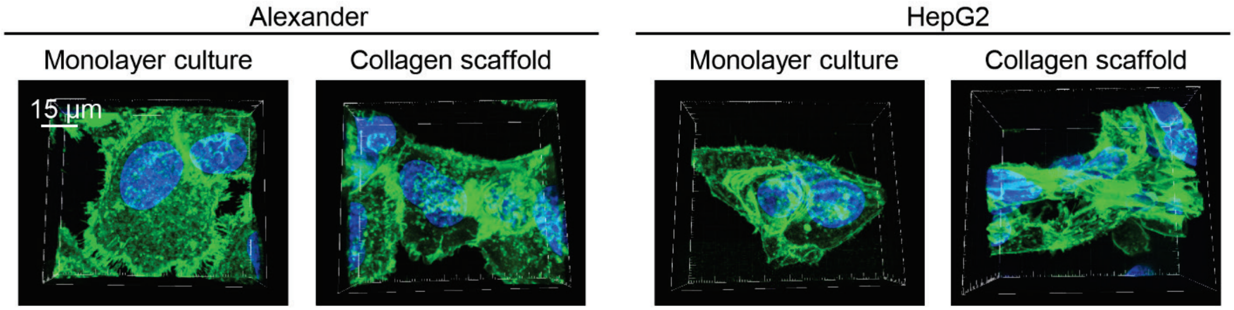

Figure S1. Morphological changes upon mechanical constrain of cells grown in 3D-engineered microenvironment. HepG2 and Alexander cells were grown either in standard monolayer culture (MC) or in collagen scaffolds (CS). Cell membranes were labeled with CellMask™ Green (green). Hoechst 33342 (blue) dye was used to counterstain nuclei. Labeled cells were then imaged by confocal microscopy. 3D reconstruction was done using open-source software Icy (https://icy.bioimageanalysis.org).

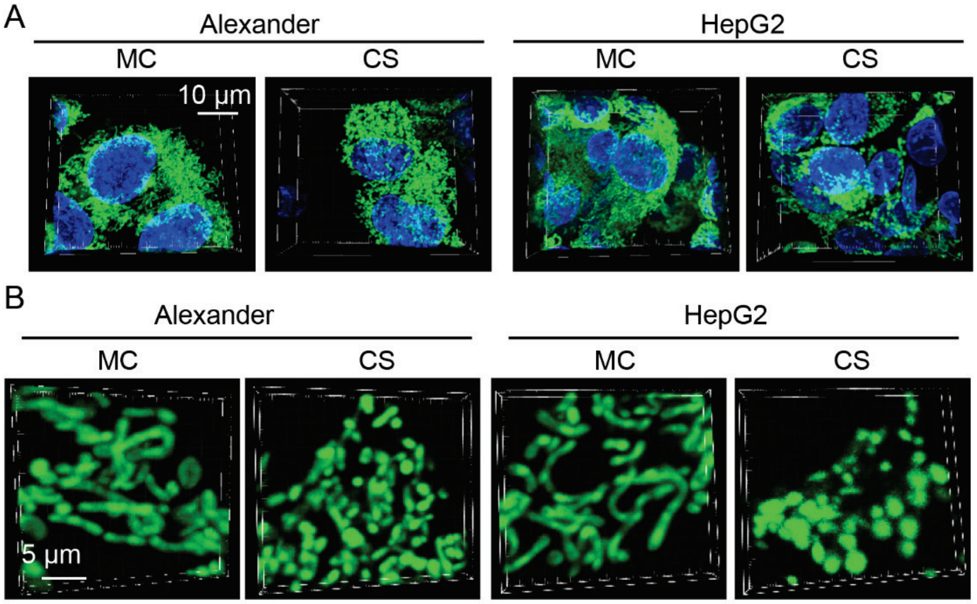

Figure S2. Alexander and HepG2 cells were cultivated for 7 days in either MC or CS conditions. Mitochondria (green) were labeled using MitoTracker® Green. Hoechst was used for nucleus (blue) as a counterstain. Labeled cells were imaged by confocal microscopy. 3D reconstruction

was done using open-source software Icy (https://icy.bioimageanalysis.org). Full cell is presented in (A) zoomed focus on single mitochondria is shown in (B).

A

LDI-FP-660-20-X-3-SM04-FU-CW	
Optical fiber	biconical
Waist diameter	15±5 μm
Protection	TiO_2 coating
Wavelenght	649 nm
Laser power	63 μW
Power density	<1 kW/cm^2
Distance	500 μm

B

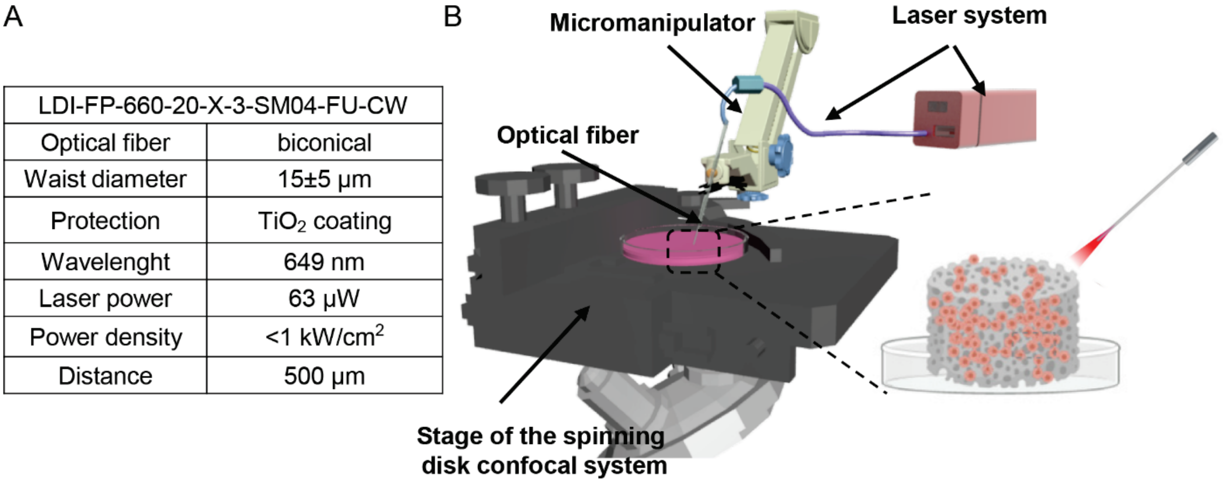

Figure S3. (A) Characterization of the HFLP laser system. (B) Experimental setup of laser irradiation of cells.

Legends for Movies

Movie S1 (separate file). 4D visualization of viability of control (untreated) Alexander cells. Alexander cells were cultivated in collagen scaffolds (CS) for 7 days. Then cells were labeled with LIVE/DEAD® Viability/Cytotoxicity Kit. Specifically, cells were loaded with calcein-AM (green) and ethidium homodimer (EthD-1, red). Hoechst 33342 was used as a counterstain for nucleus (blue). 4D images were acquired by confocal microscopy. 4D reconstruction and visualization were done using open-source software Icy (https://icy.bioimageanalysis.org).

Movie S2 (separate file). 4D visualization of viability of laser irradiated Alexander cells. Alexander cells were cultivated in collagen scaffolds (CS) for 7 days. Then cells were labeled with LIVE/DEAD® Viability/Cytotoxicity Kit. Specifically, cells were loaded with calcein-AM

(green) and ethidium homodimer (EthD-1, red). Hoechst 33342 was used as a counterstain for nucleus (blue). Labelled cells were subjected to 649 nm high fluence low-power (HFLP) laser irradiation for 90 min. 4D images were acquired by confocal microscopy. 4D reconstruction and visualization were done using open-source software Icy (https://icy.bioimageanalysis.org).

Movie S3 (separate file). 4D visualization of viability of control (untreated) HepG2 cells. HepG2 cells were cultivated in collagen scaffolds (CS) for 7 days. Then cells were labeled with LIVE/DEAD® Viability/Cytotoxicity Kit. Specifically, cells were loaded with calcein-AM (green) and ethidium homodimer (EthD-1, red). Hoechst 33342 was used as a counterstain for nucleus (blue). 4D images were acquired by confocal microscopy. 4D reconstruction and visualization were done using open-source software Icy (https://icy.bioimageanalysis.org).

Movie S4 (separate file). 4D visualization of viability of laser irradiated HepG2 cells. HepG2 cells were cultivated in collagen scaffolds (CS) for 7 days. Then cells were labeled with LIVE/DEAD® Viability/Cytotoxicity Kit. Specifically, cells were loaded with calcein-AM (green) and ethidium homodimer (EthD-1, red). Hoechst 33342 was used as a counterstain for nucleus (blue). Labelled cells were subjected to 649 nm high fluence low-power (HFLP) laser irradiation for 90 min. 4D images were acquired by confocal microscopy. 4D reconstruction and visualization were done using open-source software Icy (https://icy.bioimageanalysis.org).

Uncropped immunoblot scans

Figure 6A

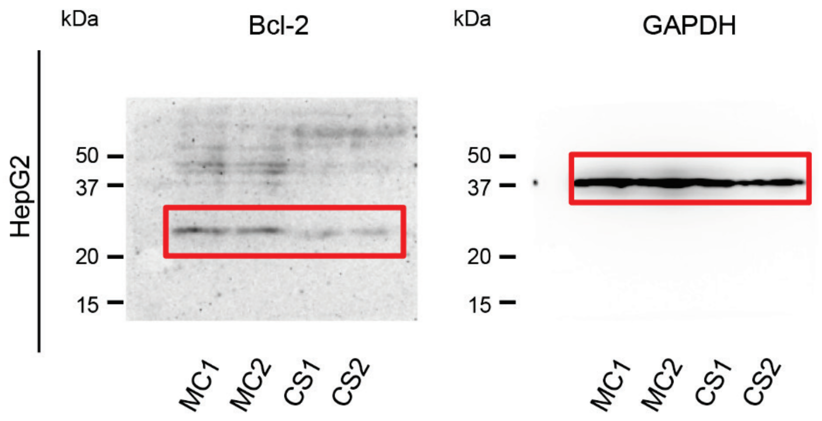

Figure 7A

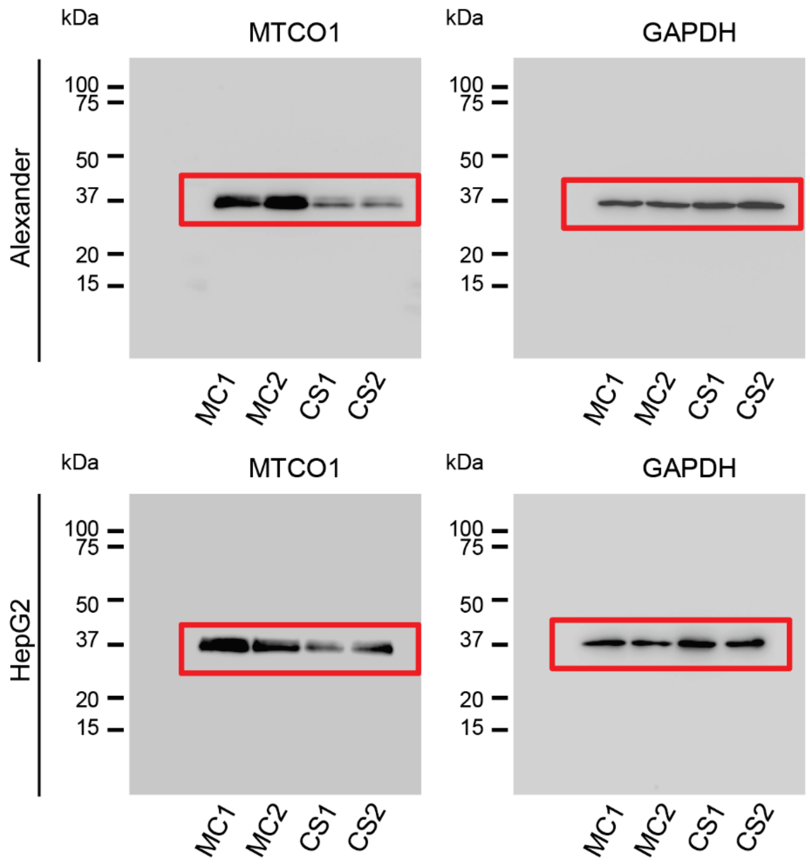

Macro Supplement Material

Macro 1

run("Split Channels");

selectWindow("C1-frame_t_0.ets - C405, C488, C561");

run("Enhance Contrast", "saturated=0.35");

selectWindow("C2-frame_t_0.ets - C405, C488, C561");

run("Enhance Contrast", "saturated=0.35");

selectWindow("C3-frame_t_0.ets - C405, C488, C561");

run("Enhance Contrast", "saturated=0.35");

run("Merge Channels...", "c1=[C1-frame_t_0.ets - C405, C488, C561] c2=[C2-frame_t_0.ets - C405, C488, C561] c3=[C3-frame_t_0.ets - C405, C488, C561] create keep");

Macro 2

selectWindow("C3-frame_t_0.ets - C405, C488, C561");

roiManager("Measure");

selectWindow("C2-frame_t_0.ets - C405, C488, C561");

roiManager("Measure");

Macro 3

run("Split Channels");

selectWindow("C1-frame_t_0.ets - C405, C488, C561");

run("Enhance Contrast", "saturated=0.35");

selectWindow("C2-frame_t_0.ets - C405, C488, C561");

run("Enhance Contrast", "saturated=0.35");

selectWindow("C3-frame_t_0.ets - C405, C488, C561");

run("Enhance Contrast", "saturated=0.35");

run("Merge Channels...", "c1=[C1-frame_t_0.ets - C405, C488, C561] c2=[C2-frame_t_0.ets - C405, C488, C561] c3=[C3-frame_t_0.ets - C405, C488, C561] create");

run("Despeckle");

run("Z Project...", "projection=[Max Intensity]");

selectWindow("MAX_frame_t_0.ets - C405, C488, C561");

setTool("rectangle");

run("Specify...", "width=512 height=512 x=843 y=603 slice=1");

Macro 4

```
selectWindow("MAX_frame_t_0.ets - C405, C488, C561");

run("Crop");

run("Split Channels");

selectWindow("C1-MAX_frame_t_0.ets - C405, C488, C561");

run("Merge Channels...", "c1=[C3-MAX_frame_t_0.ets - C405, C488, C561] c2=[C2-MAX_frame_t_0.ets - C405, C488, C561]
c3=[C1-MAX_frame_t_0.ets - C405, C488, C561] keep");

selectWindow("C2-MAX_frame_t_0.ets - C405, C488, C561");

run("RGB Color");

selectWindow("C3-MAX_frame_t_0.ets - C405, C488, C561");

run("RGB Color");

run("Multi Stack Montage...", "stack_1=[C2-MAX_frame_t_0.ets - C405, C488, C561] stack_2=[C3-MAX_frame_t_0.ets - C405,
C488, C561] stack_3=RGB stack_4=*None* rows=1 columns=3");
```
